# Disruption of the Placenta–Brain Axis in Transgenic Mice Lacking Serotonin Transporter (SERT) in Trophoblast Cells

**DOI:** 10.3390/ijms27010436

**Published:** 2025-12-31

**Authors:** David T. Ellenberger, Zhen Lyu, Rosalind T. B. Herrington, Jessica A. Kinkade, Gustavo W. Leone, Ji Ying Sze, Nathan J. Bivens, R. Frank Baker, R. Michael Roberts, Trupti Joshi, Cheryl S. Rosenfeld

**Affiliations:** 1 Department of Pathobiology and Integrative Biomedical Sciences, University of Missouri, Columbia, MO 65211, USA; 2 Christopher S. Bond Life Science Center, University of Missouri, Columbia, MO 65211, USAjoshitr@health.missouri.edu (T.J.); 3 Department of Biomedical Sciences, Joan C. Edwards School of Medicine, Marshall University, Huntington, WV 25703, USA; 4 Department of Biological Sciences, University of Missouri, Columbia, MO 65211, USA; 5 Veterinary Diagnostic Laboratory, University of Missouri, Columbia, MO 65201, USA; 6 Department of Pathology & Laboratory Medicine, Medical College of Wisconsin, Milwaukee, WI 53226, USA; 7 Department of Molecular Pharmacology, Albert Einstein College of Medicine, Bronx, NY 10461, USA; 8 Genomics Technology Core Facility, University of Missouri, Columbia, MO 65211, USA; 9 Department of Advanced Light Microscopy Core, Bond Life Sciences Center, University of Missouri, Columbia, MO 65211, USA; 10 Department of Animal Sciences, University of Missouri, Columbia, MO 65211, USA; 11 Department of Biochemistry, University of Missouri, Columbia, MO 65211, USA; 12 Department of Biomedical Informatics, Biostatistics, and Medical Epidemiology, School of Medicine, University of Missouri, Columbia, MO 65201, USA; 13 MU Institute for Data Science and Informatics, University of Missouri, Columbia, MO 65211, USA; 14 Department of Genetics Area Program, University of Missouri, Columbia, MO 65211, USA; 15 Department of Thompson Center for Autism and Neurobehavioral Disorders, University of Missouri, Columbia, MO 65211, USA

**Keywords:** trophoblast giant cells, fetus, selective serotonin reuptake inhibitors, placenta, neural development

## Abstract

Serotonin reuptake inhibitors (SSRIs) are commonly prescribed to pregnant women experiencing depression. Such drugs, however, might adversely affect placenta and fetal brain development. Parietal trophoblast giant cells (pTGCs) in the mouse placenta are postulated to internalize maternal serotonin (5-HT) via transport through SERT, encoded by *Slc6a4*, and to provide the initial source of 5-HT to the emerging brain via the placental–brain axis. Genetic deletion of *Slc6a4* in pTGCs has been hypothesized to impact placental and fetal brain development. A transgenic mouse line with high-affinity SERT, encoded by *Slc6a4*, was selectively deleted by pairing mice with Cre recombinase linked to *Prl2c2*, with LoxP sites flanking the *Slc6a4* gene. PRL2C2 is solely expressed by pTGCs and other giant cells of the placenta. To compare placental and fetal brain development in selective *Slc6a4* KO and WT mice, 5-HT content in the placenta and fetal brains of conceptuses was measured. No significant differences in 5-HT content were evident between knockout (KO) and wild-type (WT) placentas or fetal brains. However, there were significantly fewer pTGCs in KO placentas compared to WT (*p* ≤ 0.05). Sexually dimorphic differences in gene expression were evident in the placenta and fetal brain between KO and WT counterparts, with female conceptuses showing the most dramatic responses, including decrease in *Prl7a2*, *Prl5a1*, *Prl3a1*, *Slc28a3*, and *Ceacam 15* in female placental samples. These findings suggest that ablation of *Slc6a4* in pTGC disrupts the placenta–brain axis in a sex-dependent manner. The results might have important clinical ramifications for pregnant women being treated with SSRIs.

## 1. Introduction

An increasing number of pregnant women suffer from depression, with reports ranging from 7% up to 20.7% [1,2,3,4,5]. Maternal depression may adversely affect the mother’s health, as well as that of her unborn offspring. Disorders linked to maternal depression include low birth weight of offspring, preterm birth, postpartum depression, preeclampsia, and neurobehavioral deficits in the offspring [6,7,8]. Depression has been linked to serotonin (5-HT) deficiency [9,10,11].

The amount of 5-HT available to activate post-synaptic neurons is primarily regulated by the serotonin transporter (SERT) protein, encoded by the *Slc6a4* gene [12]. Approximately 13% of pregnant women are prescribed anti-depressants primarily targeting SERT, otherwise termed selective serotonin reuptake inhibitors (SSRIs), to alleviate this debilitating condition [13]. By competitively binding SERT, these drugs allow 5-HT to remain in the synaptic cleft, where this catecholamine can bind and activate 5-HT receptors in the post-synaptic neuron in various brain regions associated with emotive behaviors [14,15,16].

While SSRIs have been beneficial in treating maternal depression, they can also impact the fetal placenta–brain axis. The placenta is an ephemeral organ that facilitates nutrient and gas exchange between the mother and fetus during pregnancy. This organ helps buffer the fetus from potentially toxic and teratogenic agents [17,18,19,20], but, as an endocrine organ, it is also vulnerable to xenobiotics, including SSRIs. Animal models, in vitro cell culture studies, and human epidemiological results indicate that, besides impacting placental 5-HT levels, maternal treatment with SSRIs might influence the placenta–brain axis [21].

5-HT appears to accumulate in the human placenta [22], but it remains unclear whether it is synthesized in significant amounts by trophoblast (TB) cells [23,24], even though the machinery for its biosynthesis and catabolism has been demonstrated [25,26,27,28,29]. Disagreements about the source of placental 5-HT also exist for the mouse [30]. 5-HT immunoreactivity is visible in the ectoplacental cone [30,31], but during the emergence of the junctional zone (E9-12), it is localized largely in pTGCs, particularly those nearest decidual blood vessels [31]. An apparent concentration gradient from the pTGCs through the spongioTB has been proposed to mark the route for provisioning the fetus [32]. In this regard, there appears to be a progressive switch during brain development from an early placental source of 5-HT, which is vital for proper forebrain development, to a later endogenous source from the fetal brain itself [31,32]. That early placental source has been proposed to be either 5-HT accrued from the mother [24,31,33] and/or synthesized in the placenta at the expense of maternal tryptophan [26,30,32,34,35]. Kliman et al. [24] provide robust evidence that *Slc6a4*/SERT is the primary mediator of 5-HT transport from the maternal side to the placenta.

The TB lineage itself is initiated as the blastocyst forms. By E9.5, this junctional region comprises spongioTB and invasive secondary TGCs [36,37,38]. In the functionally mature placenta seen at E12.5, pTGCs form a narrow layer between the spongioTB and the maternal decidua and express *Prl2c2*, which encodes proliferin (PLF/PRL2C2), a protein that is exclusively expressed in the placenta. The unique expression pattern of *Prl2c2* has provided an avenue for creating Cre-based knockouts (KOs) of specific genes within these cell populations [39,40].

Placenta in whole-body *Slc64a4*/SERT KO mice are reduced in weight at embryonic age (E) 18 compared to controls and have increased apoptosis and areas of necrosis, hemorrhage, and fibrosis in the spongioTB region [28]. We found that placentas at E12.5 from *Slc64a4*/SERT KO mice have considerably increased pTGC-to-spongioTB area ratio relative to WT [41]. RNAseq analysis comparing placenta of *Slc64a4*/SERT KO to WT counterparts revealed significant elevations in expression of genes associated with intestinal functions, including nutrient sensing, uptake, catabolism, and blood clotting [41]. Integrative analyses revealed that upregulation of many of these genes was linked with pTGC layer expansion [28]. Ostensibly, the pups of whole-body *Slc64a4*/SERT KO show normal early development but display autism spectrum disorder (ASD)-like behavioral deficits as they mature [42,43,44].

Whole-body *Slc6a4* knockout (KO) mice have been invaluable in understanding the broad effects of maternal SSRI exposure. However, these models do not allow dissection of the specific pathways through which SSRIs may lead to developmental disorders. This is mainly because these models do not distinguish between the effects of SSRIs on the placenta and their direct effects on the developing fetal brain. A model that examines reduced transport of 5-HT in physiologically relevant tissue of the placenta could significantly advance our understanding of the placenta–brain axis, the biochemical and physiological communication between the placenta and the fetal brain, and its role in the development of ASD and other neurobehavioral disorders. If the inhibition of *Slc6a4*/SERT in the placenta is found to contribute significantly to these conditions, it could revolutionize how we approach prenatal care. This might include the development of new diagnostic tools to identify at-risk individuals early in pregnancy and the creation of therapeutic strategies aimed at mitigating these risks before birth, offering a proactive approach to managing and possibly preventing ASD and related conditions. In the current work, we hypothesized that selective ablation of *Slc6a4* KO in pTGCs and potentially related Spa-TGCs would affect serotonin and catecholamine concentrations in the placenta and fetal brain and lead to global gene transcription changes in both organs. Herein, we report on our creation and molecular and phenotypic characterization of *Slc6a4* KO mice selective for pTGCs and potentially Spa-TGCs and demonstrate how they might offer a promising tool in unraveling the complex pathways leading to neurobehavioral disorders following maternal SSRI exposure.

## 2. Results

### 2.1. Catecholamine Analysis

**Figure 1:**
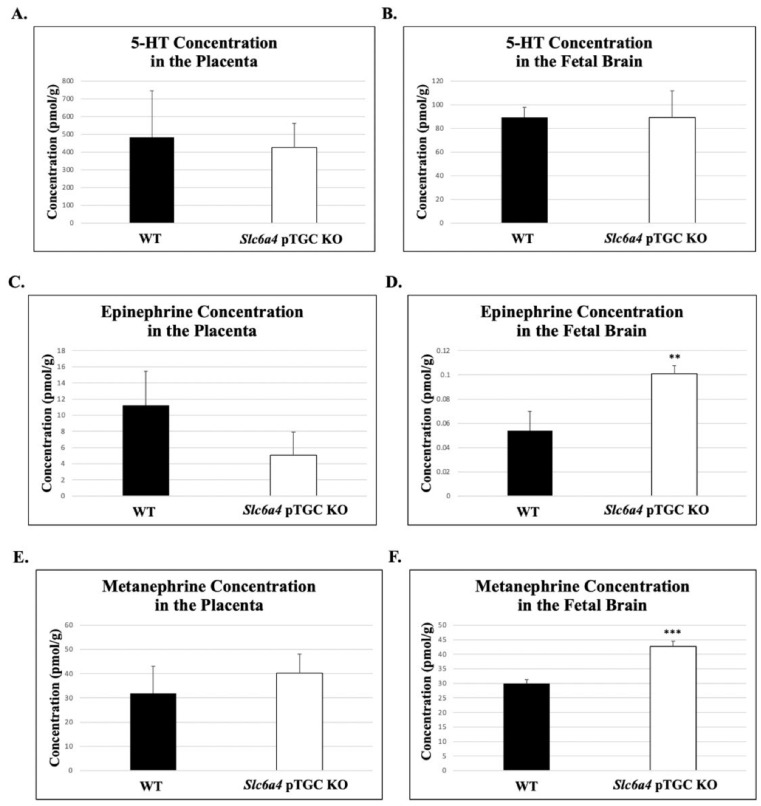
Concentrations of 5-HT, epinephrine, and metanephrine in the placenta and fetal brain of WT and S*lc6a4* pTGC KO samples. (**A**) Concentrations of 5-HT in the placenta. (**B**) Concentrations of 5-HT in the fetal brain. No significant differences in 5-HT were noted in the placenta or fetal brain. (**C**) Concentrations of epinephrine in the placenta. (**D**) Concentrations of epinephrine in the fetal brain. Epinephrine concentrations were significantly increased in the S*lc6a4* pTGC KO fetal brain. (**E**) Concentrations of metanephrine in the placenta. (**F**) Concentrations of metanephrine in the fetal brain. Metanephrine concentrations were significantly increased in the S*lc6a4* pTGC KO fetal brain. Replicates tested were as follows: three WT fetal brains, seven WT placenta, five S*lc6a4* pTGC KO fetal brains, and eleven S*lc6a4* pTGC KO placenta samples. All data are presented as mean ± SEM. ** *p* = 0.01. *** *p* = 0.003.

Based on an unpaired *t*-test, there were no significant differences in average 5-HT concentrations in *Slc6a4* pTGC KO placenta vs. WT placenta (426.34 pmol/g ± 136.0 vs. 483.55 pmol/g ± 261, respectively; *p* = 0.83, Figure 1A). Likewise, there were no differences in 5-HT concentrations in S*lc6a4* pTGC KO fetal brain vs. WT placenta fetal brain samples (89.1 pmol/g ± 22.5 vs. 89.2 pmol/g ± 8.66, respectively; *p* = 0.99, Figure 1B). With the same statistical approach, there were no significant differences in average epinephrine concentrations in *Slc6a4* pTGC KO placenta vs. WT placenta (5.07 ± 2.84 vs. 11.23 pmol/g ± 4.23, respectively; *p* = 0.23, Figure 1C).

However, *Slc6a4* pTGC KO fetal brains had a significantly higher concentration of epinephrine compared to WT fetal brain samples (0.1 pmol/g ±0.0067 vs. 0.05 pmol/g ± 0.015, respectively; *p* = 0.01, Figure 1D). There were no significant differences in average metanephrine concentrations in *Slc6a4* pTGC KO placenta vs. WT placenta (40.23 ± 7.83 vs. 31.86 pmol/g ± 11.28, respectively; *p* = 0.54, Figure 1E). However, *Slc6a4* pTGC KO fetal brains had a significantly higher concentration of metanephrine than WT fetal brains (42.67 pmol/g ± 1.88 vs. 29.9 pmol/g ± 1.34, respectively; *p* = 0.003, Figure 1F). None of the other catecholamines showed differences in the placenta or fetal brain between *Slc6a4* pTGC KO vs. WT samples (Figure S2).

### 2.2. Histological Analysis:

**Figure 2:**
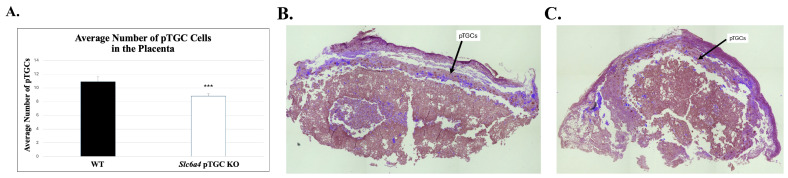
Average number of cells in the pTGC layer of the placenta for WT and *Slc6a4* pTGC KO samples. (**A**) Graphical comparison of the average number of pTGCs in WT vs. *Slc6a4* pTGC KO placental samples. (**B**) Example WT placenta with pTGCs designated. (**C**) Example *Slc6a4* pTGC KO placenta with pTGCs designated. Replicates tested were as follows: 13 samples from WT mice and 18 placenta samples from pTGC *Slc6a4* KO mice. All data are presented as mean ± SEM. *** *p* = 0.007.

While 5-HT concentrations and many catecholamines did not differ between the two genotypes based on an unpaired *t*-test, placenta from *Slc6a4* pTGC KO mice had a reduced number of pTGCs relative to WT placenta samples (8.8 ± 0.34 vs. 10.9 ± 0.72, respectively; *p* =0.007, Figure 2).

### 2.3. Immunohistochemistry for SLC6A4 in pTGC Slc6a4 KO and WT Placental Samples

**Figure 3:**
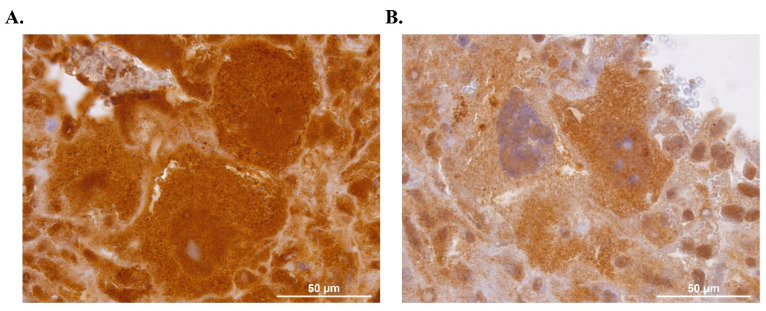
Immunohistochemistry for SLC6A4 in WT and *Slc6a4* pTGC KO placenta. (**A**) Representative WT placenta sample showing clear immunoreactivity for SL6A4 as visualized using 3,3′-diaminobenzidine (DAB, brown chromogen) in three pTGCs observed in the field of view. (**B**) Representative *Slc6a4* pTGC KO placenta showing greatly reduced immunoreactivity for Sl6A4 or decreased brown granules in three pTGCs observed in the field of view. Five WT and four pTGC *Slc6a4* KO placental samples were analyzed.

To examine the expression of SL6A4 in the pTGC *Slc6a4* KO and WT placenta, immunohistochemistry was performed with a rabbit-derived polyclonal antibody against this protein. Four pTGC *Slc6a4* KO and five WT samples were analyzed as part of these studies. As shown in Figure 3, pTGC *Slc6a4* KO had qualitatively reduced expression in the pTGC compared to WT samples. Potential explanations for such residual expression in the pTGCs of pTGC *Slc6a4* KO mice are included in the Discussion.

### 2.4. RNA Sequencing

#### 2.4.1. General Features Based on Organ, Genotype, and Sex Differences

The average number of reads across all samples was 138,151,694.6, the average number of mapped reads was 135,065,559, and the average percentage of alignment was 98.2% (Table S3). The 2D PCA plot reveals clear separation between placenta and fetal brain samples; however, clear separation was not evident based on genotype or sex (Figure S3). Volcano plot analyses revealed that at a q value ≤ 0.05 and a twofold change, 666 transcripts differed between *Slc6a4* pTGC KO and WT female placenta samples, with 332 up-regulated in WT relative to *Slc6a4* pTGC KO and 334 up-regulated in *Slc6a4* pTGC KO compared to WT samples (Figure S4A). For the male placenta samples, 40 differentially expressed genes were identified between *Slc6a4* pTGC KO and WT, with 35 up-regulated in *Slc6a4* pTGC KO and 5 up-regulated in WT samples relative to the other group (Figure S4B). No genes were differentially expressed based on genotype and sex in fetal brain samples at a q value ≤ 0.05 and a twofold change. Thus, differences were consider instead based on a *p* value ≤ 0.05 and a twofold change difference. Based on this less stringent analysis, 285 transcripts were differentially expressed in *Slc6a4* pTGC KO and WT female brain samples, with 78 up-regulated in WT and 207 up-regulated in *Slc6a4* pTGC KO samples (Figure S4C). For the male brain samples, 341 showed differential expression based on these analyses, with 98 up-regulated in *Slc6a4* pTGC KO and 243 up-regulated in WT samples (Figure S4D).

#### 2.4.2. Differentially Expressed Genes in Placenta Based on Genotype and Sex Differences

**Figure 4:**
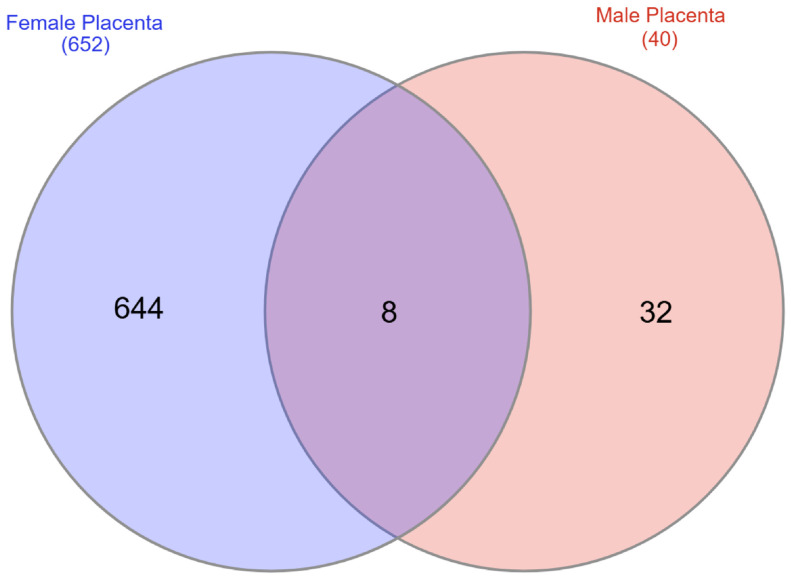
Of the 652 differentially expressed transcripts in the female placenta and the 40 differentially expressed genes in the male placenta, only 8 overlapped: *Tmem178*, *Ppbp*, *Gclc, C1s1*, *Fgl2*, *Gpr153*, *Hbb-y*, and 4732465J04Rik.

Of the 652 differentially expressed transcripts in the female placenta and the 40 differentially expressed genes in the male placenta, only 8 overlapped (Figure 4): *Tmem178*, *Ppbp*, *Gclc, C1s1*, *Fgl2*, *Gpr153*, *Hbb-y*, and 4732465J04Rik. The full list of differentially expressed genes for the female placenta is provided in Supplementary File S1, and that for the male placenta is provided in Supplementary File S2. The most up- and down-regulated transcripts based on fold change for the female placenta are shown in Table 1, and those for the male placenta are listed in Table 2. These tables also include general functions for these genes. It is noteworthy that *Slc6a4* was reduced approximately 3.5-fold (q value = 0.01) in female *Slc6a4* pTGC KO placenta relative to WT female placenta, whereas comparable differences were not detected based on genotype in the male placenta (see highlighted row in Supplementary File S1).

#### 2.4.3. Target Enrichment and Predicted Diseases/Pathways in Placenta Based on Genotype and Sex Differences

**Figure 5:**
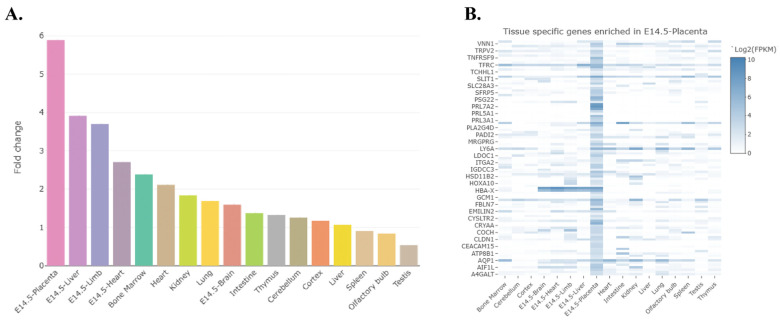
Tissue enrichment analysis of genes differentially expressed in *Slc6a4* pTGC KO vs. WT female placenta. (**A**) The differentially expressed transcripts are primarily expressed in the placenta followed by liver, extremities, heart, and bone marrow. (**B**) The heat map generated from this program reveals that many of the differentially expressed transcripts that are enriched in the placenta include several prolactin forms, including *Prl7a2*, *Prl5a1*, and *Prl3a1*, as well as *Slc28a3* and *Ceacam 15*.

**Figure 6:**
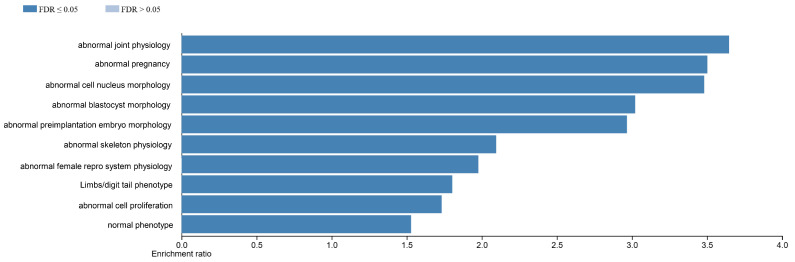
Disease processes associated with differentially expressed transcripts in the female placenta, include those associated with pregnancy complications and abnormal pre-implantation embryonic development.

The TissueEnrich program [45] was then used to determine which organs of the mouse have an abundance of transcripts for differentially expressed genes in the female and male placenta, and, in the case of the placenta, which transcripts might be exclusively expressed in this organ. For the female placenta, the differentially expressed transcripts are primarily expressed in the placenta, followed by the liver, extremities, heart, and bone marrow (Figure 5A). The heat map generated from this program reveals that many of the differentially expressed transcripts that are enriched in the placenta include several prolactin forms, including *Prl7a2*, *Prl5a1*, and *Prl3a1*, as well as *Slc28a3* and *Ceacam 15* (Figure 5B). For the male placenta, the differentially expressed transcripts are primarily associated with the embryonic liver, intestine, heart, kidney, and lung (Figure S5A). Disease processes associated with differentially expressed transcripts include those associated with pregnancy complications and abnormal pre-implantation embryonic development (Figure 6). In contrast, those associated with genes differentially expressed in the male placenta include those linked to hemorrhage, hemostasis, and coagulation (Figure S5B).

#### 2.4.4. Differentially Expressed Genes in Brain Based on Genotype and Sex Differences

**Figure 7:**
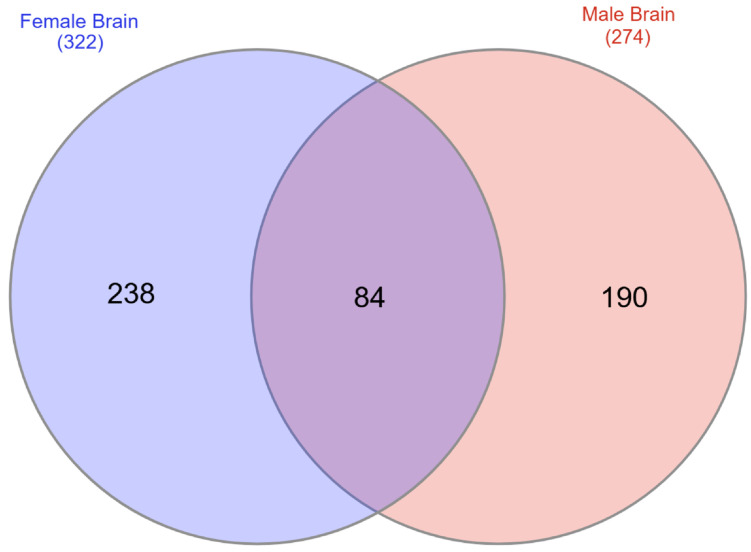
Venn diagram comparison reveals that of the 322 differentially expressed transcripts in the female brain compared to the 274 differentially expressed genes in the male brain, 84 genes overlapped. The female comparison had 238 unique transcripts, and 190 transcripts were only differentially expressed in the male comparison.

As mentioned above, no transcripts were differentially expressed in the brain based on q or adjusted *p* values alone. Thus, the following comparisons are based solely on *p* values. Venn diagram comparison revealed that, of the 322 differentially expressed transcripts in the female brain compared to the 274 differentially expressed genes in the male brain, 84 genes overlapped (Figure 7). The female comparison had 238 unique transcripts, and 190 transcripts were only differentially expressed in the male comparison. The full list of DEGs based on *p* value only for female *Slc6a4* pTGC KO vs. WT is provided in Supplementary File S3, and that for male *Slc6a4* pTGC KO vs. WT is provided in Supplementary File S4. The most up- and down-regulated transcripts based on fold change for the female brain are shown in Table 3, and those for the male brain are listed in Table 4. These tables again include general functions for these genes.

#### 2.4.5. Target Enrichment and Predicted Diseases/Pathways in Brain Based on Genotype and Sex Differences

The TissueEnrich program [45] was then used to determine which organs of the mouse have an abundance of transcripts for transcripts differentially expressed in the female and male brain. For the female brain, the differentially expressed transcripts are primarily expressed in the heart, limb, and placenta (Figure S6A). Biological processes associated with DEGs in the female brain include those involved ectoderm differentiation and cellular components (Figure S6B). For the male brain, the differentially expressed transcripts are primarily associated with the heart, lung, limb, and placenta (Figure S7A). Analogous to DEGs in the female brain, those differentially expressed in the male brain are associated with ectoderm differentiation and enzyme inhibitor activity (Figure S7B).

#### 2.4.6. Differentially Expressed Genes Based on Organ and Sex Differences

We next considered how ablation of *Slc6a4* in the pTGCs of the placenta might impact normal sex differences in this organ and in the brain. As shown in Figure S8A, the placenta of WT individuals had 494 transcripts that differed between the sexes, whereas *Slc6a4* pTGC KO only had 136 differentially expressed transcripts. Thirty-one genes overlapped between these two genotypes. For the fetal brain, we once again had to only consider *p* value. With this approach, 299 transcripts were differentially expressed between WT male vs. female fetal brain samples. In contrast, 490 genes are differentially expressed in *Slc6a4* pTGC KO male vs. *Slc6a4* pTGC KO female fetal brain samples, with 67 transcripts overlapping between the two genotypes (Figure S8B). The full list of DEGs for WT and *Slc6a4* pTGC KO placenta are shown in Supplementary Files S5 and S6, respectively. The complete list of DEGs for WT and *Slc6a4* pTGC KO placenta are shown in Supplementary Files S7 and S8, respectively.

#### 2.4.7. Target Enrichment and Predicted Diseases/Pathways Based on Organ and Sex Differences

**Figure 8:**
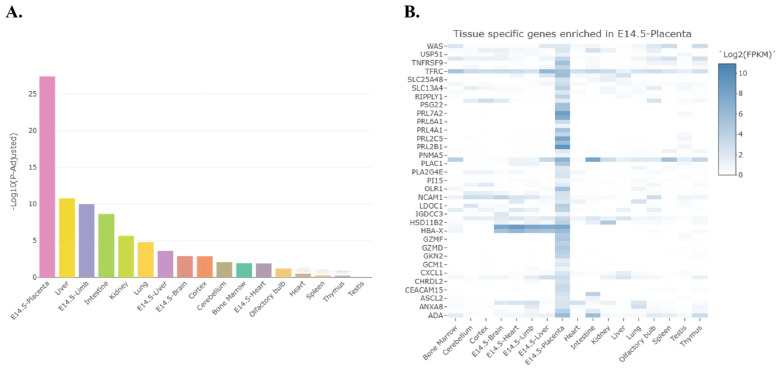
Tissue enrichment and heat map analyses for genes that show sex differences in WT placenta. (**A**) Organs enriched for DEGs in WT male vs. WT female placenta include the placenta, followed by the liver, limb, intestine, kidney, lung, liver, embryonic brain, cortex, and cerebellum. (**B**) Heat map analysis of DEGs in WT male vs. female placenta enriched in the placenta includes several prolactin transcripts (*Prl7a2*, *Prl6a1*, *Prl4a1*, *Prl2c5*, and *Prl2b1)*, two transporters (*Slc25a48* and *Slc13a4)*, and *Hsd11b2.*

Organs enriched for DEGs in WT male vs. WT female placenta include the placenta, followed by the liver, limb, intestine, kidney, lung, liver, embryonic brain, cortex, and cerebellum (Figure 8A). Heat map analysis of DEGs in WT male vs. female placenta enriched in the placenta revealed genes including several prolactins (*Prl7a2*, *Prl6a1*, *Prl4a1*, *Prl2c5*, and *Prl2b1)*, two transporters (*Slc25a48* and *Slc13a4)*, and notably *Hsd11b2* (Figure 8B). This latter enzyme catalyzes the metabolism of glucocorticoids and confers protection to the placenta against maternal forms of this stress hormone [46]. Transcript levels of *Hsd11b2* are higher in WT female placenta relative to WT male placenta.

Tissue enrichment analysis of DEGs in *Slc6a4* pTGC KO male vs. *Slc6a4* pTGC KO female placenta revealed that these transcripts are enriched in the placenta, followed by the embryonic liver, heart, embryonic limb, lung, bone marrow, kidney, and embryonic heart (Figure S9A). Heat map analysis of DEGs in the KO mice enriched in the placenta revealed a smaller number of genes compared to WT placenta (Figure S9B). Importantly, solute transporters and *Hsd11b2* do not show sexually dimorphic differences in *Slc6a4* pTGC KO male vs. *Slc6a4* pTGC KO female placenta, as evident in WT placenta (Figure 8B). The only prolactin form that shows sex differences in expression in the KO placenta is *Prl3d1.*

**Figure 9:**
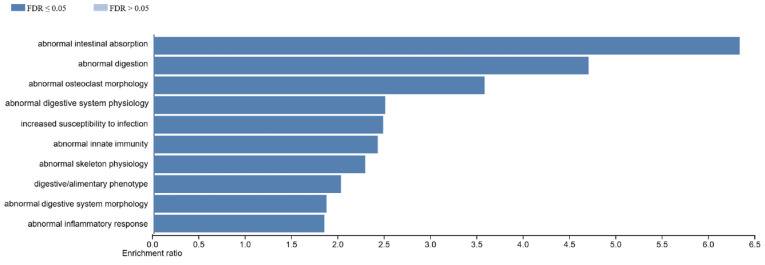
Diseases processes associated with DEG in WT male vs. WT female placenta. Phenotypic pathways/diseases associated with DEG in WT male vs. female placenta are those associated with nutrient digestion and absorption and inflammatory response pathways.

Phenotypic pathways/diseases associated with DEGs in WT male vs. female placenta are associated with nutrient digestion and absorption and inflammatory response pathways (Figure 9). In contrast, phenotypes and disease processes associated with DEGs in *Slc6a4* pTGC KO male placenta vs. *Slc6a4* pTGC KO female placenta includes those associated with abnormal embryonic development, elastic tissue changes, reproductive system changes, and mitotic abnormalities (Figure S10).

**Figure 10:**
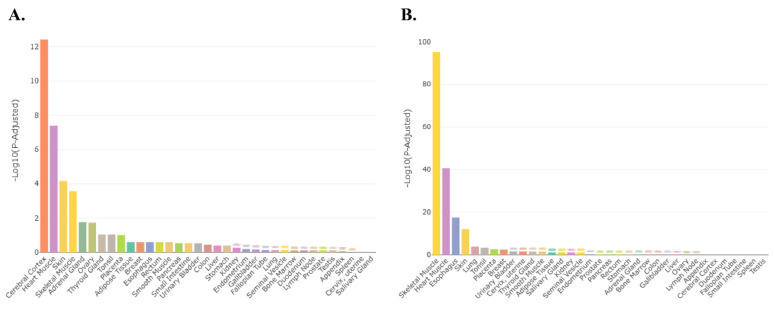
Tissue enrichment and heat map analyses for genes that show sex differences in WT and *Slc6a4* fetal brain. (**A**) WT fetal brain. Transcripts that are differentially expressed in WT male vs. female fetal brain are enriched in the cerebral cortex, followed by the heart muscle, skin, skeletal muscle, adrenal gland, and ovary. (**B**) *Slc6a4* pTGC KO fetal brain. *Slc6a4* pTGC KO male vs. *Slc6a4* pTGC KO female fetal brain, DEG are associated with skeletal muscle, heart muscle, esophagus, and skin, but surprisingly not any neural tissues.

**Figure 11:**
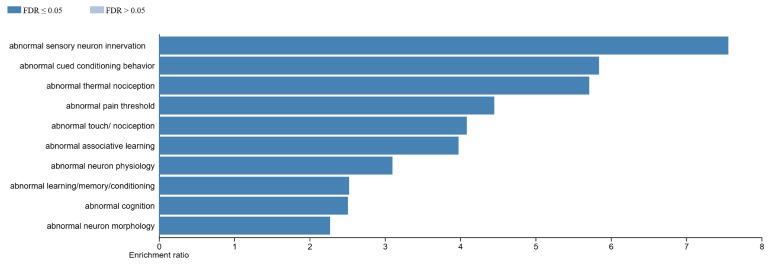
Diseases processes associated with DEGs in WT male vs. WT female fetal brain. Phenotypes and diseases associated with DEGs in WT male vs. WT female fetal brain include abnormal sensory neuron innervation, behavioral changes, anormal neuron physiology, abnormal cognition, abnormal nociception, abnormal neuron physiology and morphology, and abnormal learning and memory.

Transcripts that are differentially expressed in WT male vs. female fetal brain are enriched in the cerebral cortex, followed by the heart muscle, skin, skeletal muscle, adrenal gland, and ovary (Figure 10A). For *Slc6a4* pTGC KO male vs. *Slc6a4* pTGC KO female fetal brain, DEGs are associated with skeletal muscle, heart muscle, esophagus, and skin, but surprisingly not any neural tissues (Figure 10B). Phenotypes and diseases associated with DEGs in WT male vs. WT female fetal brain include abnormal sensory neuron innervation, behavioral changes, anormal neuron physiology, abnormal cognition, abnormal nociception, abnormal neuron physiology and morphology, and abnormal learning and memory (Figure 11). Consistent with the tissue enrichment results, DEGs in *Slc6a4* pTGC KO male vs. *Slc6a4* pTGC KO female fetal brain are linked more to skeletal muscle changes, such as abnormal I band morphology, abnormal sarcomeres, abnormal muscle fiber morphology and physiology, and impaired muscle contractility (Figure S11).

## 3. Discussion

The goal of this study was to determine how ablation of *Slc6a4* in the pTGCs of the mouse placenta might impact the placenta and fetal brain. *Slc6a4*/SERT appears to be the primary mediator of 5-HT transport from the maternal to the fetal side of the placenta [24]. In turn, the placenta appears to be integral in providing 5-HT to the developing fetal brain [31,32]. We generated Cre recombinase linked to *Prl2c2* to excise the *Slc6a4* gene, which codes for the SERT in the pTGC layer of the placenta and is known to be competitively bound by SSRIs, such that SERT cannot bind to 5-HT. We excised the *Slc6a4* gene by flanking it with LoxP sites, allowing Cre recombinase to identify the gene and remove it. The SERT knockout was localized to the pTGCs by linking Cre to *Prl2c2*, which is only expressed in pTGCs of the placenta. The hypothesis assessed herein was that 5-HT levels in the placenta and fetal brain of offspring with the *Slc6a4* gene knocked out in pTGCs, that are the primary purveyors of 5-HT to the fetal brain, would be reduced in both organs. However, 5-HT concentrations did not differ between *Slc6a4* pTGC KO compared to WT placenta and fetal brain samples (Figure 1). There are several potential explanations for these findings. Other TB cells might express SERT, allowing internalization of maternal 5-HT. The placenta is abundantly perfused with maternal and fetal blood with circulating platelets, which are known to contain abundant concentrations of 5-HT [47,48,49]. Other solute transporters might also compensate for the absence of SERT in the placenta. Candidates for such transporters include VMAT2/SLC18A2, OCT1/SLC22A1, OCT2/SLC22A2, and/or OCT3/SLC22A3 [29,50,51,52,53]. OCT3 expression by syncytiotrophoblast cells of the rat placenta is essential for 5-HT transfer, and this transporter can be suppressed by glucocorticoids [51]. A comparable situation might exist in the mouse and possibly the human placenta. Another possibility as to why 5-HT concentrations in the *Slc6a4* pTGC KO placenta and fetal brain might not have differed from those of WT is that the placenta may be able to synthesize 5-HT from the transfer of maternal tryptophan to this organ [26,30,32,34,35]. The placenta provides an early source of 5-HT for the emerging forebrain [31,32]. If the placenta can synthesize 5-HT, this finding would also then explain why 5-HT concentrations did not differ in the fetal brain between the two genotypes.

It is not clear why epinephrine and metanephrine were greater in the fetal brain of *Slc6a4* pTGC KO compared to WT individuals (Figure 1). One possibility is that deletion of this gene in the placenta affected expression of the synthesis, metabolism, or transport of these two catecholamines, both of which are derived from tyrosine. RNAseq data did not reveal any differences in enzymes regulating synthesis or metabolism, or in transporters for these two monoamines. No previous studies have examined how early exposure to SSRIs affect these monoamines in the placenta or developing brain. Further studies are needed to understand the underpinning mechanisms and determine how elevation in epinephrine and metanephrine in the fetal brain may impact neurodevelopment and later behavioral responses.

While no change in 5-HT concentrations was observed in the placenta, *Slc6a4* pTGC KO had decreased number of pTGCs compared to WT (Figure 2). These results are differ from those obtained in whole-body *Slc6a4* KO conceptuses, which had increased rather than reduced number of pTGCs [41]. The explanation for this inconsistency is unclear but may be a consequence of the total lack of *Slc6a4* expression throughout the placenta in the whole-body KO, leading to a compensatory upregulation in pTGC number. By selectively knocking out the gene in pTGCs, the normal balance of precursor TB cell differentiation to pTGCs, or their viability, may be altered. In this regard, SERT has been suggested to protect TB cells against caspase-3-independent-induced apoptosis [28].

RNAseq studies indicate that ablation of *Slc6a4* in pTGCs leads to sex-dependent differences in gene expression patterns in the placenta. Female placentas showed the most marked changes in response to deletion of *Slc6a4* in the pTGCs. Among the most up-regulated genes in the female placenta following the loss of *Slc6a4* were several prolactin forms, including *Prl7a2*, *Prl5a1*, and *Prl3a1,* whose translation products are important for placenta–maternal communication (Figure 5). Such gene expression differences were not evident in male placenta. Previously, somewhat analogous sex differences were observed in placentas for dams exposed to oxycodone, where many genes, including *Ceacam11*, *Ceacam14*, *Ceacam12*, *Ceacam13*, *Prl7b1*, *Prl2b1*, *Ctsq*, and *Tpbpa,* were increased in female placenta exposed to this drug [54]. The transporter *Slc28a3*/CNT3 (solute carrier family 28/concentrative nucleoside transporter 3) was also up-regulated in *Slc6a4* pTGC KO female placenta. It encodes for a concentrative nucleoside transporter protein that is essential for normal placenta development in humans and likely other species [55]. Given the key placental genes altered in *Slc6a4* pTGC KO female placenta, it is not surprising that predicted pathways/diseases affected include those that regulate normal embryonic development and pregnancy (Figure 6).

Differentially expressed transcripts for WT vs. *Slc6a4* pTGC KO male placenta are primarily associated with the embryonic liver, intestine, heart, kidney, and lung. Disease processes associated with DEGs in males include those linked to hemorrhage, hemostasis, and coagulation. Similar findings were noted in the whole-body knockout of *Slc6a4* [41] and are consistent with observed areas of hemorrhage in the spongioTB layer of knockout animals compared to WT [55].

Only pTGC KO female placenta showed reduced expression of the *Slc6a4* transcript (Supplementary File S1). These data might also explain why female placenta pTGC KO placenta demonstrated dramatic gene expression changes compared with WT female placenta, whereas male pTGC KO placenta had few gene expression changes (Supplementary File S2). In the original paper characterizing the *Slc6a4*^fl/fl^ mouse model, approximately 75% reduction in *Slc6a4* mRNA levels was reported in the raphe nuclei of P7 *SERT^fl/fl^*; *ePet1^Cre^*^/+^ (*SERT^RapheΔ^*). In the presence of Cre recombinase, exon 2 becomes spliced to exon 5 of the *Slc6a4* gene. Accordingly, RNAseq would likely detect spliced forms that lack an open reading frame to code for the protein.

Comparing gene transcripts in the fetal brain of *Slc6a4* pTGC KO vs. WT fetal brain revealed 322 differentially expressed transcripts in the female brain and 274 differentially expressed genes in male brain, with only 84 transcripts overlapping. The fact that deletion of *Slc6a4* in pTGCs of the placenta has marked effects on gene expression in the fetal brain is of particular note and is consistent with earlier studies from our laboratory [41,54,56] and those of others [57,58] implicating products released by TGCs in early brain development. It remains to be determined if the transcriptomic alterations are linked or even caused by changes in epinephrine or metanephrine, or due to other monoamines. Other studies have found that selective deletion of transcripts in the placenta can influence neurobehavioral patterns. For example, selective deletion of the receptor for IL-6 (IL-6Rα) in placental trophoblasts results in protection against inflammatory responses in the placenta and fetal brain induced by exposure of the pregnant mother to poly(I:C). The deletion, in turn, minimizes behavioral abnormalities and cerebellar neuropathologies otherwise observed in exposed WT offspring. [57].

Both the placenta and brain normally exhibit sexual dimorphic gene expression patterns [59,60,61,62,63,64,65]. What was surprising in the present study was that knockout of a single gene, *Slc6a4*, in a single cell type, the pTGC, appeared to affect these sexually dimorphic differences in both the placenta and brain. For example, deletion of *Slc6a4* in pTGCs reduced gene expression differences between male vs. female placenta samples (WT had 494 DEGs vs. 136 in *Slc6a4* pTGC KO), while the opposite was observed in the case of the fetal brain, where ablation of *Slc6a4* in pTGCs resulted in a greater number of gene expression differences (299 transcripts in WT compared to 490 transcripts in *Slc6a4* pTGC KO). One gene, *Hsd11b2*, which is up-regulated in WT female placentas [46], encodes the enzyme 11β-hydroxysteroid dehydrogenase type 2 (*11B-HSD2*), which is highly expressed in human syncytiotrophoblast cells of placental villi and the labyrinthine TB of the mouse placenta [66,67,68,69,70]. 11B-HSD2 catalyzes the oxidation of maternally derived cortisol into its inactive form, cortisone, before it is transferred to the fetus [66,67,68,69]. The action of 11B-HSD2 is crucial to fetal health, as downregulation of the enzyme increases fetal exposure to maternal glucocorticoids and is linked to intrauterine growth restriction, impaired fetal growth, and anxiogenic behavior [66,67,68,69,71]. Maternal prenatal stress, including depression and anxiety, downregulates the expression of 11B-HSD2 in the placenta, particularly prenatal stress experienced during the third trimester of pregnancy [72,73,74,75,76,77]. *T*he higher expression of this enzyme in the female mouse placenta might account for why male conceptuses are more vulnerable to maternal stress [78,79,80,81,82]. Loss of sex differences in this enzyme in *Slc6a4* pTGC KO placenta might result in both sexes becoming equally sensitive to maternal stress/elevated glucocorticoid levels.

Current findings reveal that DEGs in WT male vs. WT female fetal brain are associated with neural physiology and behavioral alterations (Figure 11). In contrast, DEGs for *Slc6a4* pTGC KO male vs. *Slc6a4* pTGC KO female fetal brain are linked to skeletal muscle changes (Figure S16). It is unclear how to interpret these results, especially given the different embryonic lineages of most neural cells vs. skeletal muscle (ectoderm vs. endoderm). The findings might suggest that deletion of this gene in the placenta influences a pluripotent stem cell population with the ability to differentiate into neural or skeletal muscle lineages. Further work is needed to determine how such sex differences in the fetal brain influence long-term brain functioning and behavioral traits.

Our studies support the notion that SSRI treatment of pregnant women might influence the placenta–brain axis. Other in vitro, human epidemiological, and animal experiments provide further evidence that this is the case. In vitro and ex vivo studies reveal that SSRIs disrupt various structural and hormonal properties of placental cell lines [21,83,84,85,86,87]. In rats, in utero exposure to venlafaxine reduces fetal placental weight [88]. Pregnant mothers using SSRIs have been reported to deliver lower-birthweight infants and exhibit higher rates of placental–fetal vascular malperfusion than controls [89]. Lower placental weights with reduced fetal vascular malperfusion have also been reported in human conceptuses exposed in utero to SSRIs [89], although no changes in placental vascularization were noted in an Australian cohort study [90]. DNA methylation, transcriptomic, and proteomic changes in the human placenta have been linked to maternal treatment with SSRIs [91,92,93]. Transcriptomic changes following maternal exposure also appear to be more pronounced in the placenta of sons than daughters, suggesting sexually dimorphic differences in vulnerability [93]. Protein levels for nerve growth factor (NGF), which is crucial in regulating neuronal cell growth and survival, were increased in human TB cells derived from placentas of SSRI-treated pregnant women [94].

Immunohistochemistry studies suggest reduced, but not complete, elimination of SLC6A4 in pTGCs of the placenta in presumptive *Slc6a4* pTGC KO individuals (Figure 3). It is possible that these cells continue to translate partial peptides for this protein that cross-reacts with the antibody tested. Another possibility is that *Sl6a4* is ablated in select pTGCs that express *Prl2c2*, which is linked to Cre recombinase, but continues to be expressed in cells that do not express this gene. It remains to be determined whether placentas from these mice have reduced or complete ablation of SL6A4/SERT function to bind 5-HT. Thus, limitations of the current work include the lack of a comprehensive assessment of protein expression and functional characteristics of SL6A4/SERT in the placenta, fetal brain, and other organs of *Slc6a4* pTGC KO and WT to confirm functional removal of this protein in pTGCs of the placenta but not in other organs. Further studies are needed to determine the degree in ability of this protein to bind 5-HT in the placenta and other organs in presumptive *Slc6a4* pTGC KO and WT conceptuses. It should be kept in mind that *Slc6a4* pTGC KO placentas have a reduced number of pTGCs (Figure 2), which could also impact such functional placental studies. While our studies suggest that deletion of *Sl6a4* in pTGCs can impact fetal brain transcriptome profiles, we have not yet confirmed that such changes lead to longstanding behavioral changes. Behavioral studies with juvenile and adult mice with selective ablation of *Sl6a4* in pTGCs, along with WT counterparts, are ongoing, and results from these experiments will be included in a follow-up report.

## 4. Methods and Materials

### 4.1. Creation of Mouse Model and Collection of Fetal Placenta and Brain Samples

To study the potential impact that SSRIs may have on the placenta–brain axis, a transgenic mouse model was developed in which the SERT (*Slc6a4*) was selectively knocked out in pTGCs. This KO strain was achieved by using Cre recombinase linked to *Prl2c2* to excise the *Slc6a4* gene. The *Slc6a4* gene was flanked by two LoxP sites, which allows Cre recombinase to excise the gene [95]. As shown in Figure 1 in [95], the two LoxP cassettes flank exons 3 and 4 of *Slc6a4*. In the presence of Cre recombinase, it results in the reading frame for this gene being disrupted with exon 2 spliced to exon 5. Cre recombinase was linked to *Prl2c2,* as this gene is exclusively expressed by pTGCs and other giant cells of the placenta [40]. As shown in Figure S1, the KO strain mouse was created by breeding mice expressing Cre recombinase linked to the *Prl2c2* gene with mice with LoxP sites flanking *Slc6a4*. After several rounds of breeding these two lines together, a pTGC knockout (KO) mouse model for *Slc6a4* was developed.

To collect brain and placenta samples for further analyses, *Slc6a4*^fl/fl^; *Prl2c2*^Cre/+^ (*Slc6a4* pTGC KO) mice were paired overnight with *Slc6a4*^fl/fl^; *Prl2c2*^+/+^ (WT) mice. Females were then examined for a copulatory plug, which indicated 0.5 days post-coitus (dpc). Females without copulatory plugs were re-bred until a plug was evident. Females were weighed daily and sacrificed at 12.5 dpc by American Veterinary Medical Association (AVMA) approved euthanasia methods (CO_2_ inhalation followed by cervical dislocation). Fetal placenta tissue was either frozen in liquid nitrogen or fixed in 4% (*w*/*v*) paraformaldehyde for histological analysis. Fetal brains were frozen in liquid nitrogen. Fetal tissue was collected for embryonic sexing procedures.

### 4.2. Genotype Analysis and PCR Sexing

Genotype analysis of embryonic tissues was performed by extracting DNA from fetal tissue with the Qiagen DNeasy Blood and Tissue DNA extraction kit (Cat. No. 69504; Qiagen, Germantown, MD, USA). Following DNA extraction, PCR analysis was conducted. To test whether a sample was WT or *Slc6a4* pTGC KO, we conducted PCR on the Cre recombinase gene and the *Slc6a4* (SERT) gene [56,95]. Primer sequences and expected product sizes are listed in Table S1. All primers were supplied by Integrated DNA Technologies (IDT, Coralville, IA, USA). PCR analysis was performed with Green Master Mix (Promega, Madison, WI, USA) according to the manufacturer’s recommendations. Reactions were run on 2% agarose (ThermoFisher Scientific, Waltham, MA, USA) gel containing ethidium bromide (ThermoFisher Scientific).

### 4.3. Catecholamine and 5-HT Analysis

LC–MS analysis of catecholamines (metanephrine, normetanephrine, norepinephrine, and dopamine) and 5-HT was conducted on pTGC *Slc6a4* KO fetal brain, pTGC *Slc6a4* KO placenta, WT fetal brain, and WT placenta tissues at 12.5 dpc. These analyses were performed by Creative Proteomics (Shirley, NY, USA). For catecholamine and 5-HT analysis, three *Slc6a4*^fl/fl^; *Prl2c2*^+/+^ (WT) fetal brains, seven *Slc6a4*^fl/fl^; *Prl2c2*^+/+^ (WT) placenta, five *Slc6a4*^fl/fl^; *Prl2c2*^Cre/+^ (*Slc6a4* pTGC KO) fetal brains, and eleven *Slc6a4*^fl/fl^; *Prl2c2*^Cre/+^ (pTGC *Slc6a4* KO) placenta were analyzed. 5-HT and the catecholamines metanephrine, normetanephrine, norepinephrine, and dopamine were analyzed.

In each case, serially diluted solutions (*n* = 9) were prepared as internal standards (ISs) for each of the six targeted compounds in 70% acetonitrile. Each standard compound carried six deuterium atoms. A 50 μL volume of test sample was mixed with 50 μL of IS solution and 900 μL of water. The mixture was then loaded onto a reversed-phase solid-phase extraction cartridge. After discarding the flow-through fraction under positive pressure, the retained compounds were eluted with 1 mL of methanol. The collected fraction was dried under a nitrogen gas flow, and the residue was re-constituted in 50 μL of 70% acetonitrile and mixed with 50 μL of 20 mM dansyl chloride solution and 50 μL of borate buffer. The mixtures were allowed to react at 40 °C for 60 min. After the reaction, 10 μL of each solution was injected into a UPLC-MRM/MS using a Waters Acquity UPLC system coupled to a Sciex QTRAP 6500 Plus mass spectrometer (SCIEX, Marlborough, MA, USA) with positive-ion detection. LC separation was conducted on a C18 UPLC column (2.1 × 150 mm, 1.8 μm, SCIEX) with 0.1% formic acid in water and 0.1% formic acid in acetonitrile as binary solvents for gradient elution (50% to 100% B in 15 min) at 50 °C and 35 °C. Concentrations of detected compounds were calculated by reference to internal standard values by interpolating linear regression curves of individual compounds constructed from analyte-to-internal standard peak ratios.

### 4.4. Statistical Analysis for Metabolomics Studies

Statistical significance of 5-HT and catecholamine concentrations for pTGC KO and WT samples was assessed using an unpaired *t*-test (GraphPad, Version 10.5.0, San Diego, CA, USA), and graphs were created with Microsoft Excel (version 16.99.2). Values with a *p* value of 0.05 or less were considered to be significantly different. All data are reported as average ± standard error of the mean (SEM). The number of replicates evaluated is reported in each of the figure legends.

### 4.5. Histological Analysis

Placenta samples, previously fixed with 4% paraformaldehyde (Fisher Scientific), were placed into a histological cassette (Fisher Scientific). In the cassette, the tissue was placed between two 70% ethanol-soaked sponges and processed fort sectioning. Tissues were processed on a VIP 5 tissue processor (Sakura, Torrance, CA, USA), paraffin-embedded on a Sakura Tissue-Tek Embedding Center, sectioned on an HM 355S microtome (Sakura) into 4–5 um thick sections, and placed on glass slides. They were stained with hematoxylin and eosin (H & E) on a Sakura Tissue-Tek Prisma Plus automated slide stainer.

Histological slides were imaged with a Leica Thunder Imager Model Organism stereoscope with a Leica DMC5400 20 Megapixel CMOS color camera (Leica, Buffalo Grove, IL, USA) to obtain montages of H&E-stained placental tissue slices at the University of Missouri Light Microscopy Center (University of Missouri, Columbia, MO, USA). A transmitted light base with the field aperture set to fully open and a 0.6X/0.35 NA objective were used for sample visualization. The Navigator component of the Leica LASX software Version 5.3.1 program was utilized to define tissue sections as regions of interest (ROIs), automatically acquire images across the ROIs with a motorized stage, and generate final montages after applying a shading-correction function. The montages were converted from the Leica-specific LIF format to TIFF files using the LASX native conversion function for final analysis.

### 4.6. Statistical Analysis for Histological Studies

Histological slides were analyzed with ImageJ software Version 1.54p by randomly placing ten equally sized rectangles (105,732 pixels) over the pTGC layer. Within each box, the number of pTGC cells was counted, and samples were subsequently grouped by genotype. The mean and SEM of the sample average was calculated for each group. Statistical significance was assessed by using an unpaired *t*-test (GraphPad, Version 10.5.0, San Diego, CA, USA), and graphs were created with Microsoft Excel (version 16.99.2). Statistically significant samples had a *p* value ≤ 0.05. In total, 18 placenta samples from pTGC Slc6a4 KO mice and 13 samples from WT mice were analyzed.

### 4.7. Statistical Analysis for Histological Studies

Briefly, dewaxed and rehydrated histologic sections were pretreated with 3% H_2_O_2_ for 15 min prior to heat-induced antigen retrieval using Diva Decloaker (DV2004, Biocare Medical, Pacheco, CA, USA). Slides were incubated in this solution for 120 °C for 10 min and then at 90 °C for 10 s. Then, the slides were treated with Background Sniper (BS966, Biocare Medical) for nonspecific protein blocking. Slides were incubated at room temperature with arabbit anti-SLC6A4 polyclonal antibody (PA589657, Invitrogen, Carlsbad, CA, USA) at a 1:100 dilution. The rabbit EnVision+ system (K4003, DAKO, Carpinteria, CA, USA) was used for antigen detection and incubated on the slides for 30 min. Immunoreactivity for this protein was visualized using 3,3′-diaminobenzidine (DAB) (K346711, DAKO) for 10 min. CAT hematoxylin counterstain (CATHE, Biocare Medical) was used for counterstain, and slides were incubated in this solution for 3 min. Slides were visualized with a Leica DM5500 B Microscope and Leica LAS V4.12 software program (Leica, Heerbrugg, Switzerland). Four pTGC *Slc6a4 KO* and five WT placental samples were subjected to immunohistochemistry against SL6A4 and analyzed.

### 4.8. RNA Isolation from Fetal Placenta and Brain Samples

To characterize changes in gene expression patterns in WT and *Slc6a4* pTGC KO conceptuses, total RNA was extracted using the Qiagen AllPrep DNA/RNA/miRNA Universal Kit (Cat. No. 80224; Qiagen, MD, USA) in accordance with the manufacturer’s protocol. The number of replicates is listed in Table S2. RNA concentration was initially assessed with a NanoDrop ND-1000 spectrophotometer (Nano Drop Technologies, Wilmington, DE, USA) and further evaluated for integrity with the Fragment Analyzer system (University of Missouri Genomics Technology Core, Columbia, MO, USA). Only samples with an RNA integrity number (RIN) score ≥ 7.0 were selected for RNA sequencing (RNAseq).

### 4.9. Illumina TruSeq RNA Llibrary Preparation and Sequencing

Libraries were constructed following the manufacturer’s protocol with reagents supplied in the Illumina mRNA Stranded Library Preparation Kit and sequenced at the University of Missouri Genomics Technology Core. In brief, mRNA was captured with oligo(dT) magnetic beads, eluted, fragmented, and primed for cDNA synthesis. cDNA was synthesized as hexamer-primed RNA fragments and reverse transcriptase. After second-strand cDNA synthesis, pre-index anchors were ligated to the cDNA ends. A subsequent PCR step was used to selectively amplify anchor-ligated DNA fragments and add indexes and primer sequences for cluster generation. Amplified cDNA constructs were purified by the addition of Axyprep Mag PCR Clean-up beads (Fisher Scientific).

Final library size was evaluated on a Fragment Analyzer (Agilent Technologies, Inc., Santa Clara, CA, USA), quantified with a Qubit fluorometer by means of the Qubit dsDNA HS Assay kit (Invitrogen), and diluted according to Illumina’s standard sequencing protocol. Libraries were pooled and run on an Illumina NovaSeq X sequencer (Illumina, San Diego, CA, USA) with a paired-end 100 bp read format on a 10 B flow cell to generate ~100 million paired reads per sample. The actual number of reads obtained for each sample is listed in Table S3.

### 4.10. RNAseq Data Processing and Bioinformatics Analyses

RNAseq data analysis pipeline started with quality assessment of raw Illumina sequencing reads by FastQC. High-quality reads were then aligned to the mouse reference genome (GRCm39) using the Hisat2 aligner with its index building functionality. Following alignment, transcript assembly and quantification were conducted by means of Cufflinks, which estimates gene expression levels in terms of FPKM (fragments per kilobase of transcript per million mapped reads). Differential expression analysis was performed using Cuffdiff (Cufflinks suite, v2.2.1). To account for multiple hypothesis testing, Cuffdiff applies the Benjamini–Hochberg procedure, which adjusts *p* values to generate q values representing the False Discovery Rate (FDR). This approach controls for false positives across the thousands of genes evaluated simultaneously. For placenta samples, differentially expressed genes (DEGs) were defined as those with log_2_ fold change (log2FC) ≥ 1 and q value < 0.05. For brain tissue, DEGs were defined as those with log2FC ≥ 1 and *p* value < 0.05. Data visualization, including volcano plots and principal component analysis (PCA), was performed with Python software Version 3.14.2 (Python Software Foundation, Fredericksburg, VA, USA) by using matplotlib and seaborn. PCA was conducted with the scikit-learn package.

### 4.11. Enrichment and Network Analyses

Enrichment analyses were carried out with TissueEnrich [45] based on the mouse ENCODE dataset [96] and EnrichR [97] with the KEGG_2019_Mouse database.

For functional enrichment analysis, DEGs were imported into the web-based GEne SeT AnaLysis Toolkit (WebGestalt) 2019 version, and searched for gene ontology molecular function (GO MF) and gene ontology biological processes (GO BP) [98].

## 5. Conclusions

In conclusion, our data show that selective deletion of *Slc6a4* in pTGCs of the mouse placenta caused sex-dependent changes in the placenta, including reduced number of pTGCs and transcriptomic alterations in the placenta that were most pronounced in the placentas of female offspring. Importantly, ablation of this gene in the placenta affected the placenta–brain axis, as evidenced by alterations in epinephrine and metanephrine in the fetal brain and sexually dimorphic gene expression changes in this organ. Ostensibly, the latter results are the most striking as it suggests discrete changes in the placenta, which could be genetic in origin or induced in response to shifts in the maternal environment, can impact early fetal brain development and likely increase long-term risk for neurobehavioral disorders, although this possibility was not directly tested in the current work. This research was conducted to address a major gap in our understanding of the potential impacts on the placenta–brain axis in women prescribed SSRIs, which target *Slc6a4*/SERT to combat depression. Our future endeavors include examining the neurobehavioral patterns in juvenile and adult mice that were devoid of *Slc6a4* expression in pTGCs of the placenta. These studies will presumably guide clinical usage of SSRIs in pregnant women and clarify potential outcomes of such drugs on the placenta-brain axis and long-term health outcomes in children exposed to such drugs while in utero.

## Figures and Tables

**Table 1:** Top five most up- and down-regulated transcripts in the female placenta WT vs. *Sl6a4* pTGC KO, with a *p*-adj (q value) ≤ 0.05.

Gene Symbol	Fold Change (WT vs. *Sl6a4* pTGC KO)	*p*-adj (q Value)	General Function of the Encoded Protein
Most Up-Regulated Transcripts			
*C1ql2*	17.15360619	0.002955	Encodes a protein that acts as a synaptic organizer at mossy fiber-CA3 synapses in the hippocampus.
*Muc2*	9.603098663	0.002955	Encodes a component of the mucous barrier in the gut lumen.
*Slc7a14*	8.211694752	0.047026	Amino acid transporter protein expressed in skin fibroblasts, neural tissue, and primary endothelial cells.
*Il23r*	7.815293207	0.026117	Forms a subunit for the receptor IL23A/IL23.
*Cldn11*	7.084863216	0.002955	Component of tight junctions in oligodendrocytes of the central nervous system.
Most Down-Regulated Transcripts			
*Galk1*	−0.49958	0.042783	Enzyme that phosphorylates galactose to galactose-1-phosphate in the Leloir pathway.
*Srsf1*	−0.4946	0.042783	Encodes a serine/arginine-rich splicing factor.
*Rian*	−0.48759	0.024938	Encodes a long non-coding RNA that is involved in chromatin organization.
*Xpo1*	−0.48572	0.024938	Encodes a protein that mediates leucine-rich nuclear export signal-dependent protein transport.
*Plac8*	−0.4826	0.009259	Predicted to enable chromatin binding activity, brown fat cell differentiation, and response to cold.

**Table 2:** Top five up- and down-regulated transcripts in the male placenta WT vs. *Sl6a4* pTGC KO with a *p*-adj (q value) ≤ 0.05.

Gene Symbol	Fold Change (WT vs. *Sl6a4* pTGC KO)	*p*-adj (q Value)	General Function of the Encoded Protein
Most Up-Regulated Transcripts			
*Rps7-ps3*	8.167531	0.027646	No available data on this gene.
*Ppbp*	4.553608	0.027646	Encodes a platelet-derived growth factor part of the chemokine family.
Most Down-Regulated Transcripts			
***Maob***	**−0.47047**	**0.027646**	**Encodes monoamine oxidase B, a mitochondrial enzyme that breaks down monoamines, including 5-HT (serotonin) in the CNS and peripheral tissues.**
*Gclc*	−0.46023	0.027646	Encodes catalytic subunit of glutamate–cysteine ligase involved in the synthesis of glutathione.
*Cldn2*	−0.44035	0.045239	Belongs to the claudin protein family and encodes a tight junction protein that forms channels in epithelial cells.
*Ctsc*	−0.44008	0.027646	Encodes cathepsin C, a lysosomal cysteine protease involved in activating serine proteases in immune cells.
*Hnf4a*	−0.4264	0.045239	Encodes a nuclear transcription factor that controls expression of hepatocyte nuclear factor 1 alpha.

**Table 3:** Top five most up- and down-regulated transcripts in the female brain WT vs. *Sl6a4* pTGC KO with a *p* value ≤ 0.05.

Gene Symbol	Fold Change (WT vs. *Sl6a4* pTGC KO)	*p* Value	General Function of the Encoded Protein
Most Up-Regulated Transcripts			
*Gh*	71.68552853	5.00 × 10^−5^	Enables growth hormone receptor binding activity.
*Afp*	63.28887421	5.00 × 10^−5^	Encodes alpha-fetoprotein produced by the yolk sac and liver during fetal life.
*Apoa4*	48.6008621	0.0076	Plasma protein involved in the regulation of lipid and glucose metabolism.
*Pcp2*	29.62904046	0.00045	Enables guanyl-nucleotide exchange factor activity and acts upstream of G-protein-coupled opsin signaling pathway.
*Cga*	24.2217299	0.00025	
Most Down-Regulated Transcripts			
*Dsp*	−0.49516	0.0333	Encodes a protein that anchors intermediate filaments to desmosomal plaques.
*Tnnt1*	−0.48167	0.04035	Encodes a protein that is a subunit of troponin that regulates striated muscle contraction.
*Rbm24*	−0.47986	0.02295	Involved in negative regulation of cytoplasmic translation, positive regulation of cell differentiation, and regulation of mRNA metabolic processes.
*Zdbf2*	−0.47788	0.02435	Encodes DBF4-type zinc finger domains.
*Ramp1*	−0.47689	0.04165	Encodes a member of the RAMP family of single-transmembrane-domain proteins and is required to transport calcitonin receptor-like receptor.

**Table 4:** Top five most up- and down-regulated transcripts in the male brain WT vs. *Sl6a4* pTGC KO with a *p* value ≤ 0.05.

Gene Symbol	Fold Change (WT vs. *Sl6a4* pTGC KO)	*p* Value	General Function of the Encoded Protein
Most Up-Regulated Transcripts			
*Prss27*	8.870774	0.00655	Encodes a tryptic serine protease that is mainly expressed in the pancreas.
*Krt13*	5.495497	0.00055	Encodes keratin, an intermediate filament protein expressed in the suprabasal layers of non-cornified stratified epithelia.
*Ppbp*	4.553608	5 × 10^−5^	Encodes a platelet-derived growth factor that stimulates the formation and secretion of plasminogen activator and acts as a chemoattractant of neutrophils.
*Tekt1*	3.16479	0.00515	Highly expressed in the testis, and in mice, this gene was localized to the spermatocytes and may play a role in spermatogenesis.
*Slc6a1*	3.020296	0.0059	It is a gamma-aminobutyric acid (GABA) transporter that localizes to the plasma membrane.
Most Down-Regulated Transcripts			
*Llcfc1*	0.158686	0.04005	Predicted to be involved in the fusion of sperm to egg.
*Ghrl*	0.168688	0.0065	Encodes the ghrelin–obestatin preproprotein that is cleaved into ghrelin and obestatin.
*Cyp24a1*	0.17729	0.04795	Encodes a cytochrome P450 enzyme that initiates the degradation of 1,25-dihydroxyvitamin D3.
*Cyp3a13*	0.19536	0.0312	Predicted to enable caffeine oxidase activity and steroid hydroxylase activity.
*Adgra1*	0.209972	0.00365	Encodes a protein that belongs to the adhesion family of G-protein-coupled receptors involved in the sensory system and blood pressure regulation.

## Data Availability

RNA sequencing data have been deposited in the Gene Expression Omnibus under accession number GSE304347 (https://www.ncbi.nlm.nih.gov/geo/query/acc.cgi?acc=GSE304347). Accessed on 29 December 2025.

## Supplementary Materials

The following supporting information can be downloaded at: https://www.mdpi.com/article/10.3390/ijms27010436/s1.
