# Supplementary material for: Disruption of the Placenta–Brain Axis in Transgenic Mice Lacking Serotonin Transporter (SERT) in Trophoblast Cells
Source: Int J Mol Sci. 2025 Dec 31;27(1):436. doi: 10.3390/ijms27010436 (PMC12787258; doi:10.3390/ijms27010436)
Supplement: Supplementary file 1 [file ijms-27-00436-s001.zip › supplementary material.pdf]

Supplementary Material

Disruption of the Placenta-Brain Axis in Transgenic Mice Lacking Serotonin Transporter (SERT) in Trophoblast Cells

David T. Ellenberger[1] Zhen Lyu[2,11], Rosalind T.B. Herrington[1,3], Jessica A. Kinkade[4], Gustavo W. Leone[5], Ji Ying Sze[6], Nathan J. Bivens[7], R. Frank Baker[8], R. Michael Roberts[9,10], Trupti Joshi[2,11,12,13], and Cheryl S. Rosenfeld[1,13,14,15]

Departments of:
[1]Pathobiology and Integrative Biomedical Sciences. University of Missouri, Columbia, Missouri, 65211, USA
[2]Christopher S. Bond Life Science Center, University of Missouri, Columbia, MO, 65211, USA
[3]Biological Sciences, University of Missouri, Columbia, Missouri, 65211, USA
[4]Veterinary Diagnostic Laboratory, University of Missouri, Columbia, Missouri, 65201, USA
[5]Department of Pathology & Laboratory Medicine, Medical College of Wisconsin, Milwaukee, Wisconsin, 53226, USA
[6]Department of Molecular Pharmacology, Albert Einstein College of Medicine, Bronx NY 10461, USA
[7]Department of Genomics Technology Core Facility, University of Missouri, Columbia, Missouri, 65211, USA
[8]Advanced Light Microscopy Core, Bond Life Sciences Center, University of Missouri, Columbia, Missouri, 65211 USA
[9]Animal Sciences, University of Missouri, Columbia, Missouri, 65211, USA
[10]Biochemistry, University of Missouri, Columbia, Missouri 65211, USA
[11]Department of Biomedical Sciences, Joan C. Edwards School of Medicine, Marshall University, Huntington, West Virginia, USA
[12]Department of Biomedical Informatics, Biostatistics, and Medical Epidemiology, School of Medicine, University of Missouri-Columbia, MO, 65201, USA
[13]MU Institute for Data Science and Informatics, University of Missouri, Columbia, Missouri, 65211, USA
[14]Department of Genetics Area Program, University of Missouri, Columbia, Missouri, 65211, USA
[15]Department of Thompson Center for Autism and Neurobehavioral Disorders, University of Missouri, Columbia, Missouri, 65211. USA

Short Title: Placenta-Brain Axis in Mice Lacking Placental Serotonin Transporter.

Keywords: Trophoblast Giant Cells; Fetus; Selective Serotonin Reuptake Inhibitors; Placenta; Neural Development

Correspondence: joshitr@marshall.edu or rosenfeldc@missouri.edu

Table S1. Primer sequences and expected product sizes/interpretation for the Cre, *Slc6a4*, and *Sry* genes.

Gene	Forward Primer	Reverse Primer	Expected Product Size/Interpretation
Cre	5’ GCG GCA TGG TGC AAG TTG AAT 3’	5’ CGT TCA CCG GCA TCA ACG TTT 3’	Cre +: 232 bp<br>Cre -: no band
Slc6a4	5’ ACT CCA GTA GAC GAT GTC AGA T 3’	5’ TAG TCA GGA AGA GTA AGG GGG 3’	WT: 467 bp<br>Heterozygous: 467 bp and 613 bp<br>Homozygous : 613 bp
Sry	5’ TCA TGA GAC TGC CAA CCA CAG 3’	5’ CAT GAC CAC CAC CAC CAC CAA 3’	Male: 440 bp<br>Female: no band

Table S2. Number of replicates tested for placenta and fetal brain RNA samples analyzed with RNAseq analysis for each genotype.

Organ tested with RNAseq Analysis	*Slc6a4* pTGC KO Male	WT Male	*Slc6a4* pTGC KO Female	WT Female
**Fetal Placenta**	4	4	4	4
**Fetal Brain**	4	3	4	4

Table S3. Number of raw reads, mapped reads, and % mapped reads for RNA seq results from placenta and fetal brain from *Slc6a4* pTGC KO and WT samples.

Sample	Genotype and Sex	Fetal Organ	Raw Reads	Mapped Reads	% of Mapped Reads
1084-R3-PRNA	WT Female	Placenta	139950812	137403466	98.2
2040-L2-PRNA	WT Female	Placenta	152167738	149449927	98.2
2040-R2-PRNA	WT Female	Placenta	151261064	148289124	98
2010-R7-PRNA	WT Female	Placenta	137623620	135118447	98.2
1048-L1-PRNA	WT Male	Placenta	143975046	141506908	98.3
1083-R2-PRNA	WT Male	Placenta	140041738	136730705	97.6
1103-L1-PRNA	WT Male	Placenta	140104178	137126576	97.9
2010-R4-PRNA	WT Male	Placenta	157371186	154416168	98.1
1103-L2-PRNA	KO Female	Placenta	130723408	128072323	98
2010-R3-PRNA	KO Female	Placenta	106459770	104224611	98
2031-L1-PRNA	KO Female	Placenta	149093062	146645110	98.3
38-L1-PRNA	KO Female	Placenta	151469266	148700063	98.2
1083-R3-PRNA	KO Male	Placenta	136156878	132966335	97.7
1103-R2-PRNA	KO Male	Placenta	143016598	140302367	98.1
2010-R1-PRNA	KO Male	Placenta	142260112	139476128	98
2010-R5-PRNA	KO Male	Placenta	154241302	151131683	98
1053-L1-FH	WT Female	Brain	136781342	134556288	98.4
1053-L2-FH	WT Female	Brain	133221372	131122422	98.4
1103-R1-FH	WT Female	Brain	125394856	123514210	98.5
2040-L2-FH	WT Female	Brain	114712810	112877488	98.4
1048-L1-FH	WT Male	Brain	100806982	98838646	98
1103-L1-FH	WT Male	Brain	128360186	126338880	98.4
2039-L1-FH	WT Male	Brain	132501004	130526902	98.5
1103-L2-FH	KO Female	Brain	150666126	148439161	98.5
2031-L1-FH	KO Female	Brain	144311700	142165972	98.5
2031-R1-FH	KO Female	Brain	162238390	159641054	98.4
2039-R3-FH	KO Female	Brain	127788688	125788400	98.4
1053-R2-FH	KO Male	Brain	142740646	140535596	98.5
1103-R2-FH	KO Male	Brain	133246946	131269499	98.5
2036-L1-FH	KO Male	Brain	130787388	128889533	98.5

2039-L2-FH	KO Male	Fetal Head	143228318	120968338	98.4
**Average**			**138151694.6**	**135065559**	**98.2**

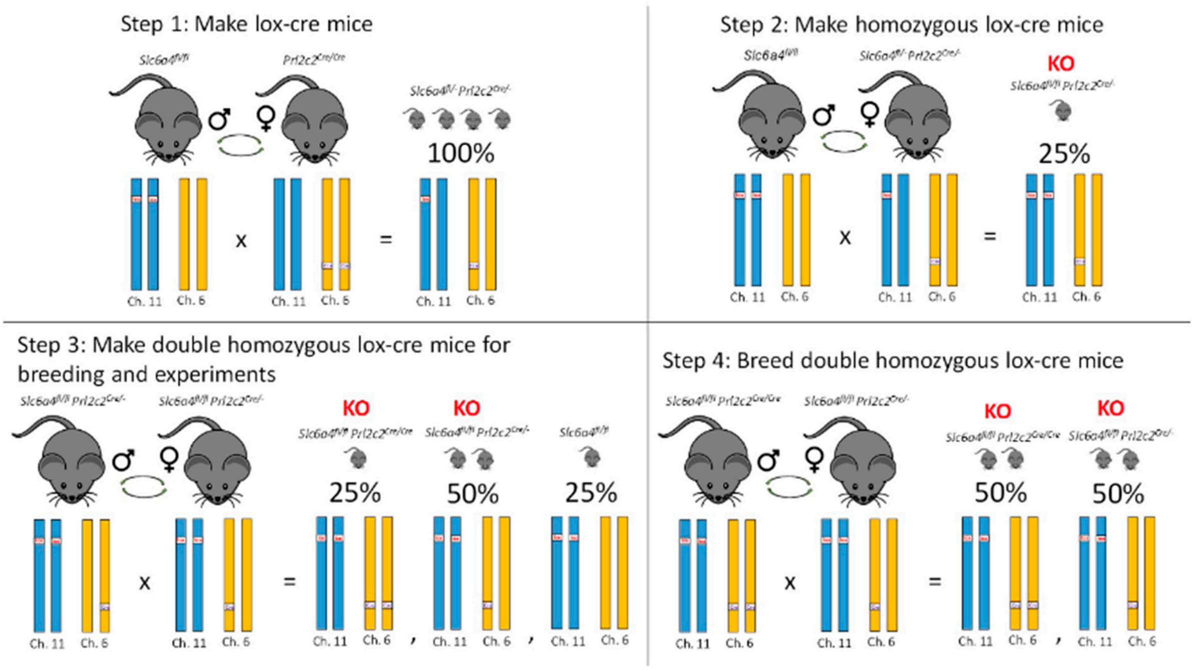

Figure S1. Breeding scheme to produce pTGC/Spa-TGC knockout for *Slc6a4*.

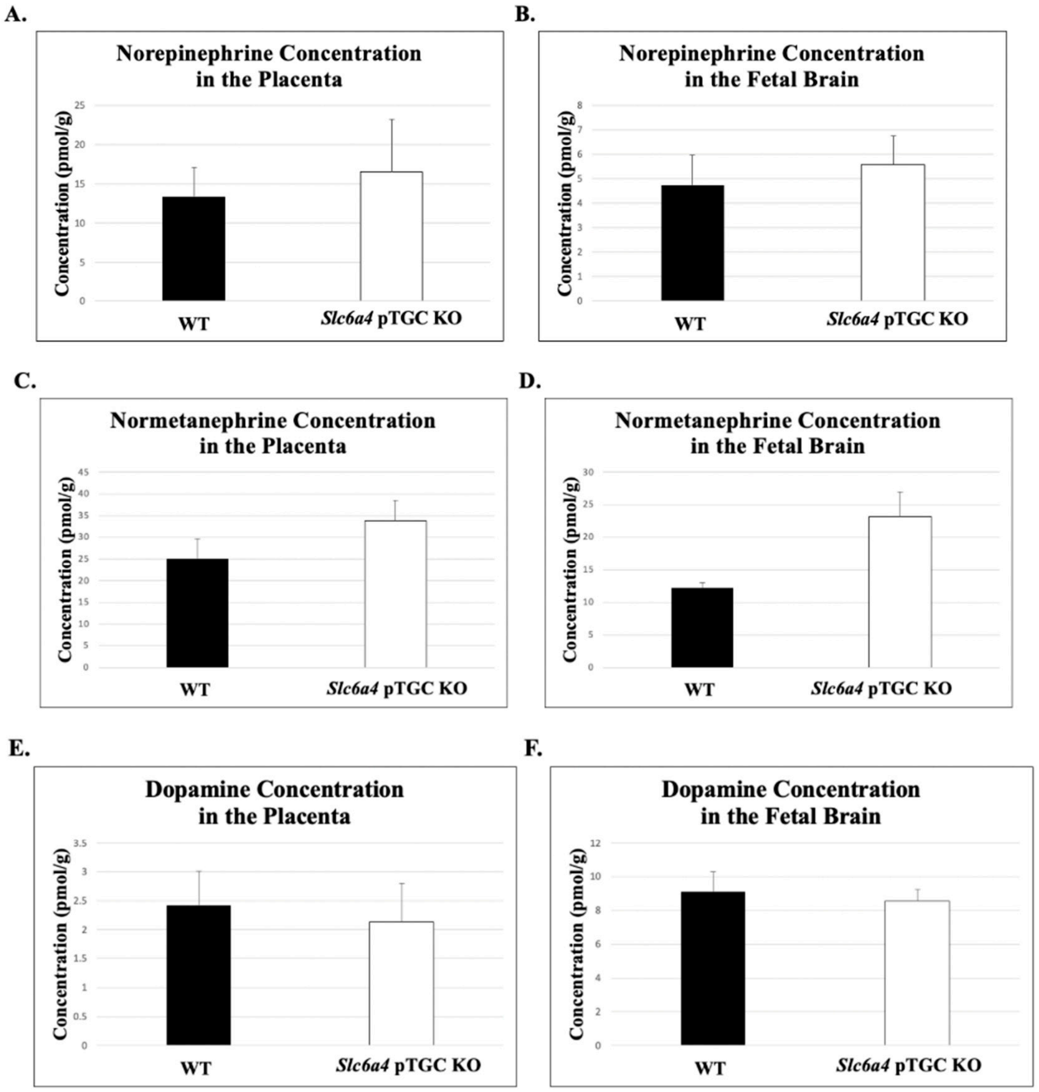

Figure S2. Concentrations of other catecholamines in placenta and fetal brain. A) There were no significant differences in average norepinephrine concentrations in *Slc6a4* pTGC KO placenta vs. WT placenta (16.58 pmol/g ± 6.65 vs. 13.41 pmol/g ± 3.68., respectively; $p = 0.73$). B) Likewise, there were no differences in norepinephrine concentrations in S*lc6a4* pTGC KO fetal brain vs. WT fetal brain samples (5.59 pmol/g ± 1.14 vs. 4.72 pmol/g ± 1.26, respectively; $p = 0.64$). C) There were no significant differences in average normetanephrine concentrations in *Slc6a4* pTGC KO placenta vs. WT placenta (33.75 pmol/g ± 4.78 vs. 25.09 pmol/g ± 4.55., respectively; $p = 0.24$). D. There were no differences in normetanephrine concentrations in S*lc6a4* pTGC KO fetal brain vs. WT fetal brain samples (23.21 pmol/g ± 3.80 vs. 12.19 pmol/g ± 0.87, respectively; $p = 0.07$. E) There were no significant differences in average dopamine concentrations in *Slc6a4* pTGC KO placenta vs. WT placenta (2.13 pmol/g ± 0.67 vs. 2.42 pmol/g ± 0.59., respectively; $p = 0.77$). F) There were no differences in dopamine concentrations in S*lc6a4* pTGC KO fetal brain vs. WT fetal brain samples (8.57 pmol/g ± 0.69 vs. 9.11 pmol/g ± 1.18, respectively; $p = 0.68$. All data are presented as mean ± SEM.

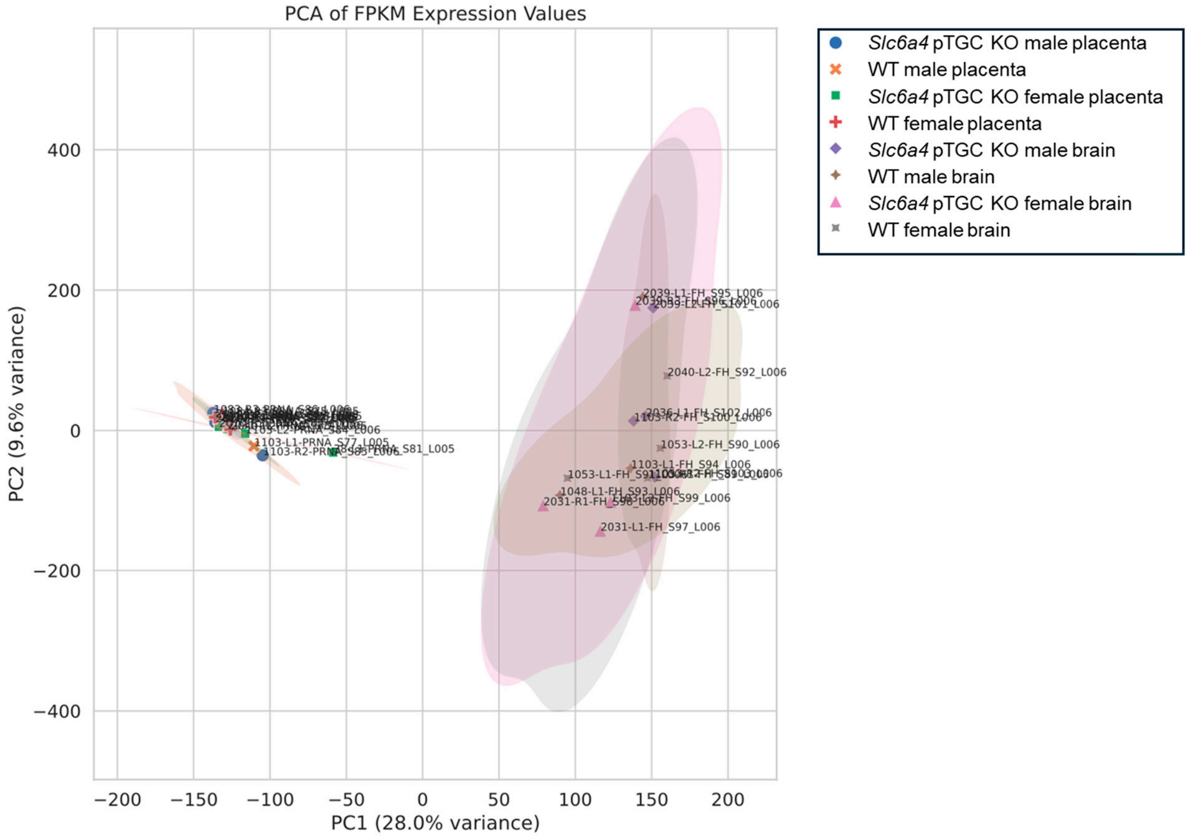

Figure S3. 2D PCA plot of *Slc6a4* pTGC KO and WT placenta and fetal brain samples. The 2D PCA plot reveals clear separation between placenta and fetal brain samples. However, clear separation was not evident based on genotype or sex.

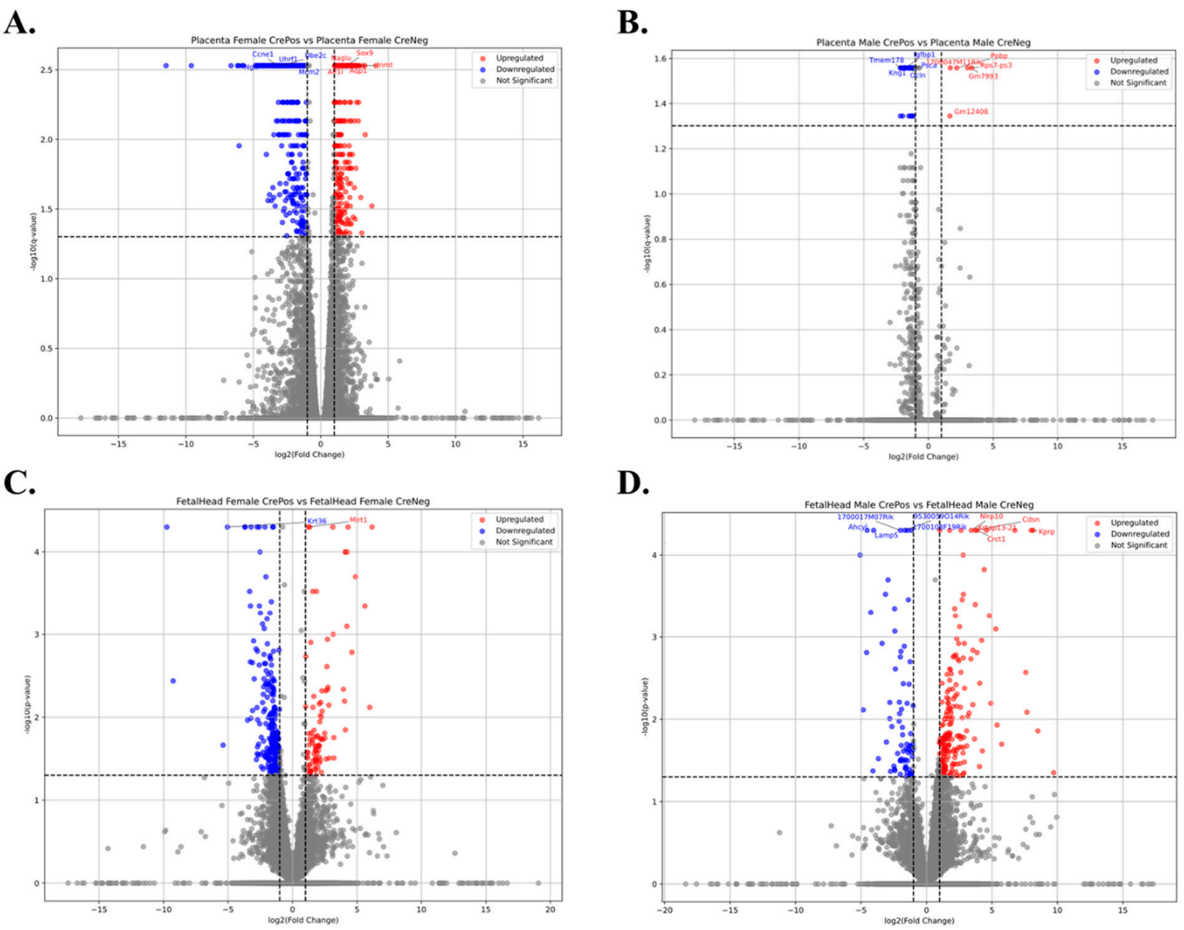

Figure S4. Volcano plot analyses for pTGC KO and WT placenta and fetal brain samples based on fetal sex. A) Female placenta samples. Volcano plot analysis revealed that at q value ≤ 0.05 and 2-fold change difference, 666 transcripts differed between *Slc6a4* pTGC KO and WT female placenta samples with 332 upregulated in WT relative to *Slc6a4* pTGC KO and 334 upregulated in *Slc6a4* pTGC KO compared to WT samples. B) Male placenta samples. For male placenta samples, 40 differentially expressed genes were identified between *Slc6a4* pTGC KO and WT with 35 upregulated in *Slc6a4* pTGC KO and 5 upregulated in WT relative to the other group No genes were differentially expressed based on genotype and sex for fetal brain samples with a q value ≤ 0.05 and 2-fold change difference. Thus, we instead consider differences based on p value ≤ 0.05 and 2-fold change difference. C) Female fetal brain samples. Based on this less stringent analysis, 285 transcripts were differentially expressed for *Slc6a4* pTGC KO and WT female brain samples with 78 upregulated in WT and 207 upregulated in *Slc6a4* pTGC KO. D) Male fetal brain samples. For male brain samples, 341 showed differential expression based on these analyses with 98 upregulated in *Slc6a4* pTGC KO and 243 upregulated in WT.

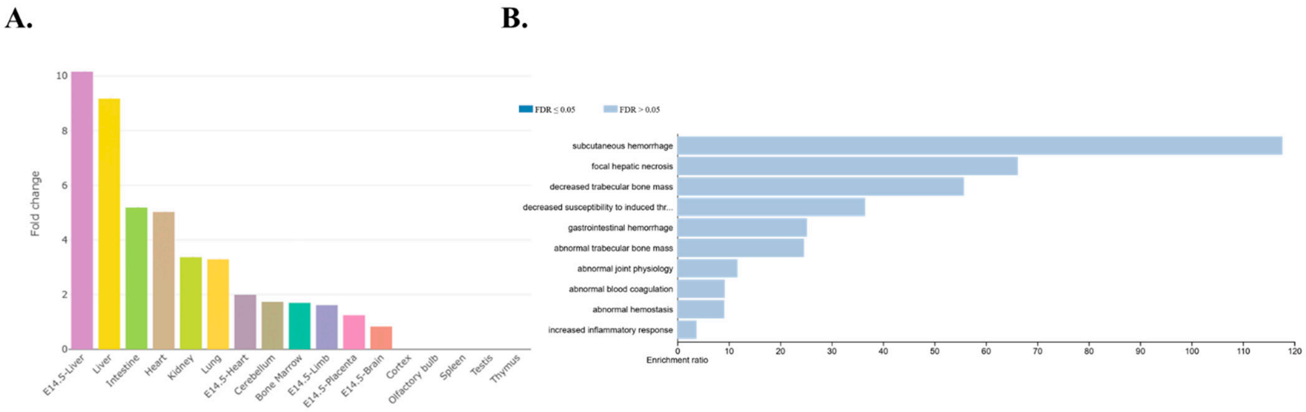

Figure S5. Tissue enrichment and phenotypic analyses of differentially expressed genes in WT vs. *Slc6a4* pTGC KO male placenta. A) Tissue enrichment analysis. For male placenta, the differentially expressed transcripts are primarily associated with embryonic liver , liver, intestine, heart, kidney, and lung. B) Phenotypic analysis. Disease processes associated with genes differentially expressed in male placenta include those linked with hemorrhage, hemostasis, and coagulation.

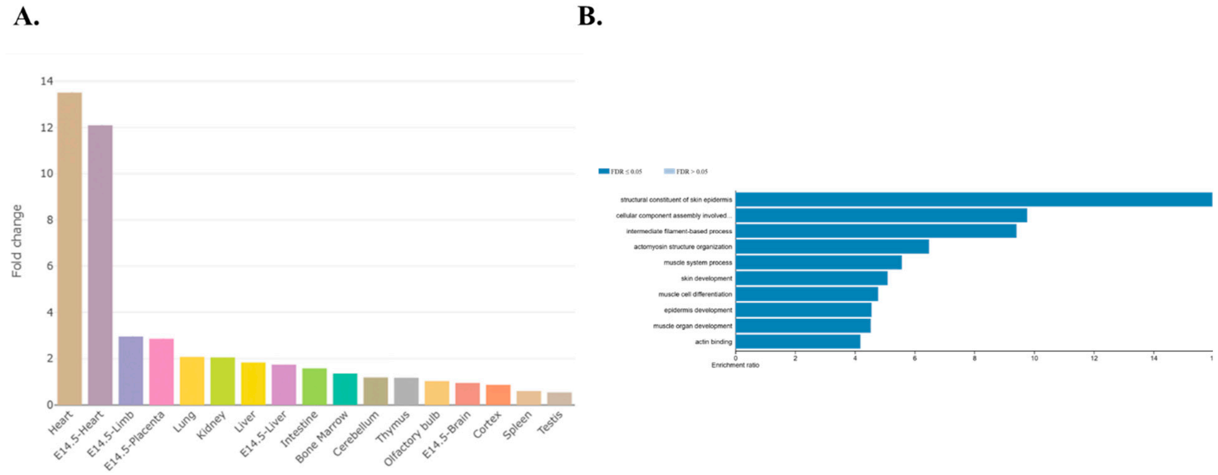

Figure S6. Tissue enrichment and biological processes associated with DEG in female fetal brain samples for WT vs. *Sl6a4* pTGC KO. A) The TissueEnrich program [62] was used to determine which organs of the mouse have an abundance of transcripts for transcripts differentially expressed in female and male brain. For the female brain, the differentially expressed transcripts are primarily expressed in the heart, limb, and placenta. B) Biological processes associated with DEG genes in the female brain include ones involved ectoderm differentiation and cellular components.

A. **B.**

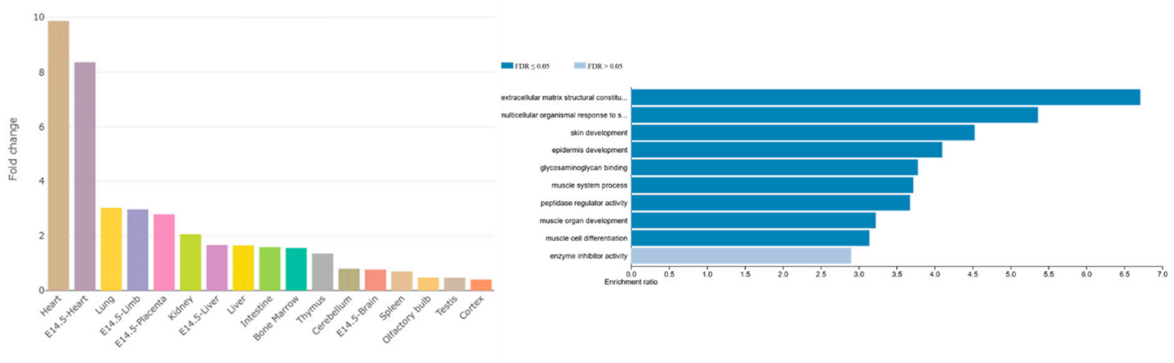

Figure S7. Tissue enrichment and biological processes associated with DEG in male fetal brain samples for WT vs. *Sl6a4* pTGC KO. A) For male placenta, the differentially expressed transcripts are primarily associated with heart, lung, limb, and placenta. B) Similar to DEG in female brain, those differentially expressed in male brain are associated with ectoderm differentiation, as well enzyme inhibitor activity.

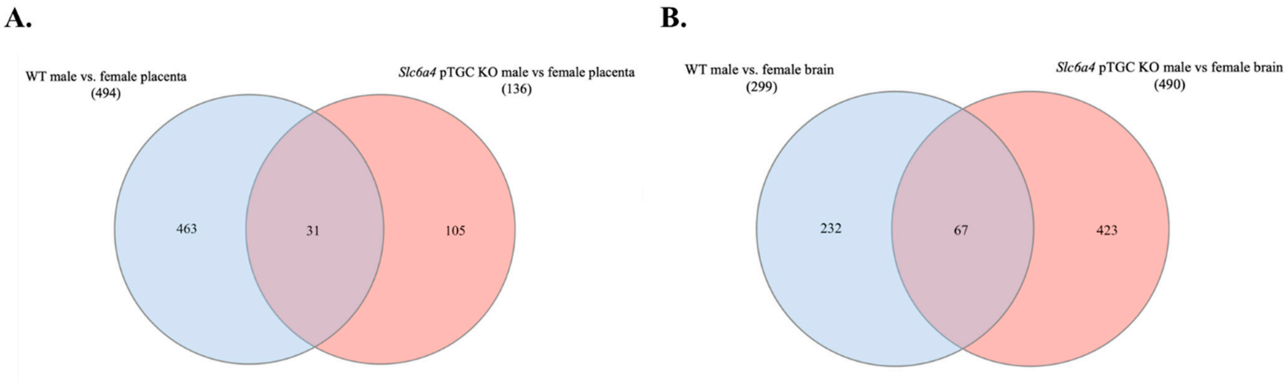

Figure S8. Venn diagram comparison of transcripts that show sex differences in WT vs. *Slc6a4* pTGC KO placenta and fetal brain samples. A) Placenta samples. The placenta of WT individuals have 494 that are different between the sexes, whereas *Slc6a4* pTGC KO only have 136 differentially expressed transcripts. Thirty-one genes overlap between these two genotypes. B) Fetal brain samples. With this approach, 299 transcripts are differentially expressed between WT and *Slc6a4* pTGC KO based on p value. In contrast 490 genes are differentially expressed in *Slc6a4* pTGC KO with 67 transcripts overlapping between the two genotypes.

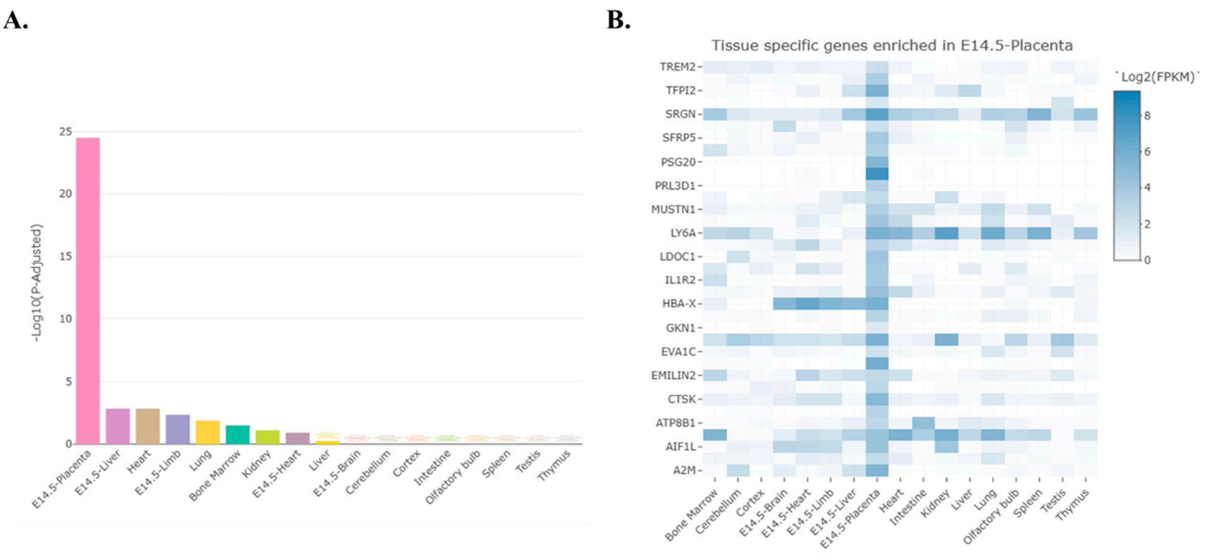

Figure S9. Tissue enrichment and heatmap analyses for genes that show sex differences in WT placenta. A) Tissue enrichment of DEG in *Slc6a4* pTGC KO male vs. *Slc6a4* pTGC KO female placenta reveals that such transcripts are enriched in the placenta followed by the embryonic liver, heart, embryonic limb, lung, bone marrow, kidney, and embryonic heart. B) Heat map analysis of DEG in the *Slc6a4* pTGC KO mice that are enriched in the placenta reveal a smaller number of genes compared to WT.

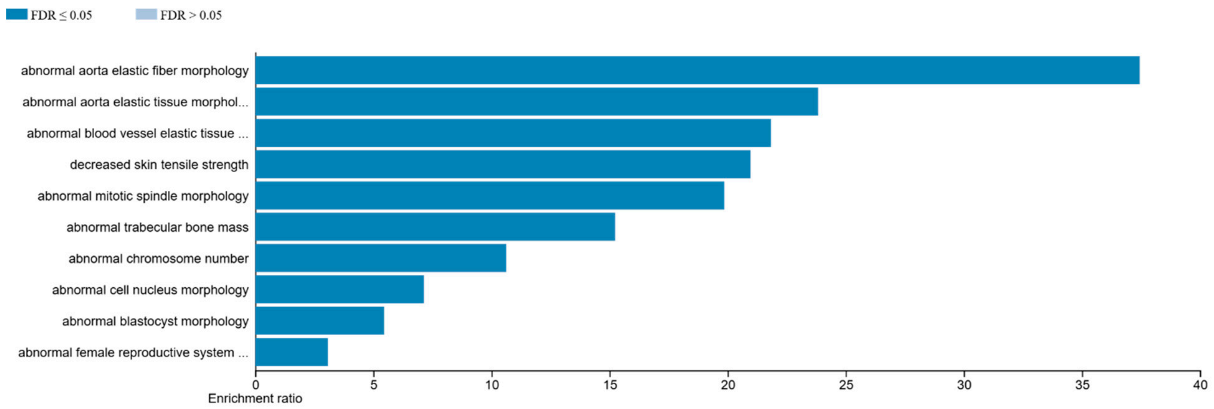

Figure S10. Diseases processes associated with DEG in *Slc6a4* pTGC KO male vs. *Slc6a4* pTGC KO female placenta. Phenotypes and disease processes associated with DEG in *Slc6a4* pTGC KO males vs. *Slc6a4* pTGC KO female placenta includes those associated abnormal embryonic development, elastic tissue changes, reproductive system changes, and mitotic abnormalities.

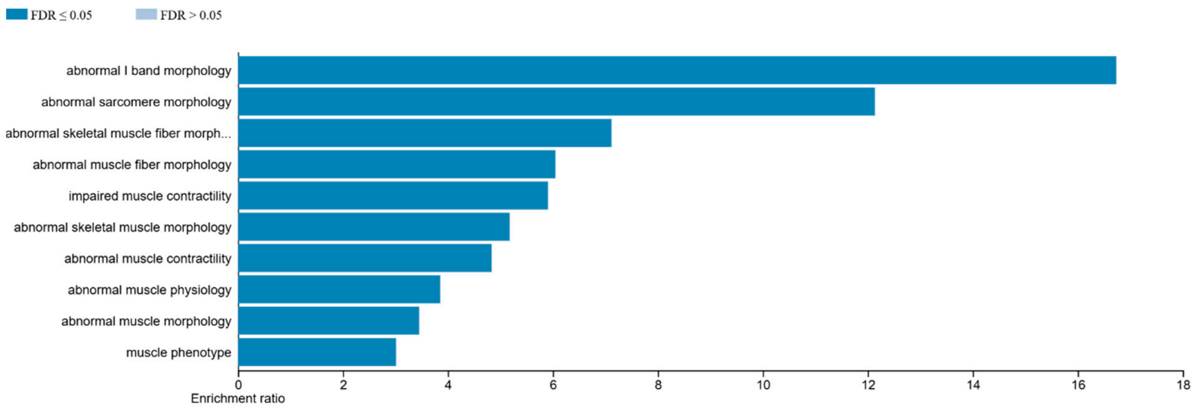

Figure S11. Diseases processes associated with DEG in *Slc6a4* pTGC KO male vs. *Slc6a4* pTGC KO female fetal brain. Differentially expressed genes in *Slc6a4* pTGC KO male vs. *Slc6a4* pTGC KO female fetal brain are linked more to skeletal muscle changes, such as abnormal I band morphology, abnormal sarcomeres, abnormal muscle fiber morphology and physiology, and impaired muscle contractility.
